# Downregulation of miR-199a-3p mediated by the CtBP2-HDAC1-FOXP3 transcriptional complex contributes to acute lung injury by targeting *NLRP1*

**DOI:** 10.7150/ijbs.37133

**Published:** 2019-09-08

**Authors:** Zhi Chen, Wei-Hua Dong, Qiang Chen, Qiu-Gen Li, Zhong-Min Qiu

**Affiliations:** 1 Department of Pulmonary and Critical Care Medicine, Tongji Hospital, Tongji University School of Medicine, Shanghai 200065, China.; 2 Department of Critical Care Medicine, Jiangxi Provincial People's Hospital Affiliated to Nanchang University, Nanchang 330006, Jiangxi, China.; 3 Department of Pulmonary and Critical Care Medicine, Jiangxi Provincial People's Hospital Affiliated to Nanchang University, Nanchang 330006, Jiangxi, China

**Keywords:** Acute lung injury, microRNA, miR-199a-3p, CtBP2, NLRP1, FOXP3

## Abstract

Emerging evidence indicates that microRNAs (miRNAs) play fundamental roles in the pathogenesis of multiple diseases, including acute lung injury (ALI). Here, we discovered that miR-199a-3p was significantly downregulated in ALI lung tissues using a microarray analysis. *In vitro* lipopolysaccharide (LPS) treatment of the human epithelial cell line A549 and the human macrophage cell line U937 caused a decrease of miR-199a-3p. Mechanically, miR-199a-3p specifically bound to the 3'-untranslated region (3'-UTR) of *NLRP1* (nucleotide-binding oligomerization domain, leucine-rich repeat and pyrin domain-containing protein 1), a critical member of inflammasomes. Ectopic overexpression or downregulation of miR-199a-3p resulted in the repression or induction of *NLRP1*, respectively, thereby downregulating or activating its downstream events. Moreover, transcription factor FOXP3 (forkhead box P3) was able to specifically bind to the promoter of miR-199a-3p. Knockdown or overexpression of *FOXP3* resulted in a decrease or induction miR-199a-3p expression, respectively. Using immunoprecipitation (IP), mass spectrometry and co-IP assays, we found that FOXP3 formed a transcriptional complex with HDAC1 (histone deacetylase 1) and CtBP2 (C-terminal-binding protein 2). Collectively, our results suggested that the CtBP2-HDAC1-FOXP3 transcriptional complex (CHFTC) could specifically bind to the promoter of miR-199a-3p and repress its expression. Downregulation of miR-199a-3p eliminated its inhibition of *NLRP1*, causing activation of NLRP1 and cleavage of pro-IL-1β and pro-IL-18 mediated by Caspase-1. The secretion of IL-1β and IL-18 further aggravated the inflammatory response and resulted in the occurrence of ALI.

## Introduction

Acute lung injury (ALI) and its more severe form, acute respiratory distress syndrome (ARDS), are common clinical syndromes associated with lung injury resulting in high mortality (30-40%) 1, 2. Multiple causes, including dysfunction of epithelial and endothelial barriers, sepsis, inhalation of harmful substances, and severe pneumonia, can result in the occurrence of ALI/ARDS. Studies have shown that activations of multiple signaling pathways are involved in the pathogenesis of ALI 3, 4. One of the most fundamental pathways is Toll-like receptor (TLR)-mediated signaling 5, 6. The pattern recognition receptors (PRRs) on the membrane of immune cells (e.g., dendritic cells, macrophages, monocytes, neutrophils and epithelial cells) distinguish pathogen-associated molecular patterns (PAMPs) and damage-associated molecular patterns (DAMPs) 5, 6. This recognition process is mediated by transmembrane proteins known as TLRs, especially TLR4 5, 6. The interactions between TLRs and their specific PAMPs mediate the myeloid differentiation primary response 88 (MyD88)-dependent pathway, whose activation further triggers NF-κB (*nuclear factor* kappa-light-chain-enhancer of activated B cells) and MAPK (*mitogen-activated protein kinase*) pathways, eventually resulting in the release of pro-inflammatory cytokines such as tumor necrosis factor α (TNF-α), interleukin (IL)-1β, IL-6 and IL-8 5, 6. Lung tissue injury causes the accumulation of glucosaminoglycan hyaluronan, which can interact with both TLR4 and TLR2 in a MyD88-dependent manner to initiate inflammatory responses in ALI 7, 8. Due to their significant changes in the progression of ALI, some proinflammatory cytokines (e.g., TNF-α, IL-1β, IL-6, IL-8, and IL-18) have been suggested as potential biomarkers for predicting morbidity and mortality in ALI/ARDS 9,10. The maturation and secretion of some pro-inflammatory cytokines, such as IL-1β and IL-18, require inflammasome activation, which is a critical event in the innate immune system 9,10. Inflammasomes are multimeric protein complexes that consist of Caspases, NLRPs (nucleotide-binding oligomerization domain, leucine-rich repeat and pyrin domain-containing proteins), and ASC (apoptosis-associated speck-like protein containing a CARD) 11, 12. NLRP1 is the first inflammasome to be discovered and characterized 11, 12. It consists of NLRP1, ASC, caspase-1 and caspase-5 11, 12. NLRP1 protein has a CARD domain in its C-terminal extension, which is required for the direct interaction between NLRP1 and pro-caspase-1 11, 12. The assembly of NLRP1 inflammasome activates Caspase-1 to cleave pro-IL-1β (pro-interleukin 1β) and pro-IL-18 13-15. The secretion of mature IL-1β and IL-18 aggravates inflammation response 13-15.

MicroRNAs (miRNAs), a class of noncoding RNAs with an average length of 22 nucleotides, play important roles in the regulation of gene silencing through different mechanisms, including (1) cleavage of the target mRNA; (2) destabilization of the target mRNA through the poly(A) tail; and (3) binding to the 3'-untranslated region (3'-UTR) of the target mRNA to disrupt the translation process 16,17. Numerous miRNAs have been identified in different biological processes 16, 17. Recent studies also found that a variety of miRNAs derived from mouse were involved in the pathogenesis of ALI/ARDS 18-21. In a lipopolysaccharide (LPS)-induced mouse ALI model, Cai and colleagues found that miR-214 and miR-451 were significantly induced, while miR-16, miR-23a, miR-24, miR-181a, miR-181b, and miR-199a were dramatically downregulated 22. Among these miRNAs, miR-16 can target the 3'-UTRs of both *IL-6* and *TNFA* 22. Another study revealed that downregulation of miR-181a could protect mouse from LPS-induced ALI by targeting *BCL2* (B-cell lymphoma 2), a regulator of apoptosis 23. Using an *in vitro* assay in pulmonary microvascular endothelial cells and an *in vivo* ALI model, Fang and colleagues found that miR-1246 could target *ACE2* (angiotensin-converting enzyme 2) and that downregulation of miR-1246 reduced cell apoptosis but increased the production of IL-1β and TNF-α 24. Although multiple miRNAs are involved in the pathogenesis of ALI, the mechanism underlying its dysregulation remains unknown. Moreover, the difficult collection of human ALI tissue samples hampers the investigation of differentially expressed miRNAs and their roles in human.

To identify miRNAs that are involved in the pathogenesis of ALI in human, we collected lung tissues from 24 ALI patients who were treated but died, and had signed organ donation consent when they were alive. With three ALI samples, we conducted a microarray analysis to measure the aberrantly expressed miRNA profile. A total of 106 differentially expressed miRNAs were identified in all three ALI samples. Then, we focused our investigation on revealing the upstream regulatory mechanism and downstream targets of miR-199a-3p, the most obviously downregulated miRNA. Our results indicated that a CtBP2-HDAC1-FOXP3 transcriptional complex (CHFTC)-dependent mechanism was responsible for the downregulation of miR-199a-3p. The inflammasome component *NLRP1* was a direct target of miR-199a-3p. Inhibition of miR-199a-3p level eliminated its repression against *NLRP1*, thereby causing the upregulation of *NLRP1*. The increased *NLRP1* enhanced the production of IL-1β and IL-18 through a Caspase-1 dependent mechanism. Elevated levels of IL-1β and IL-18 aggravated inflammatory response and resulted in the occurrence of ALI.

## Materials and Methods

### Cell lines and cell culture

The human epithelial cell line A549 (#CCL-185), the human monocyte cell line U937 (#CRL-1593), and the human microglia cell line HMC3 (CRL-3304) were purchased from the American Type Culture Collection (ATCC, Manassas, VA, USA). A549 cells were cultured in Dulbecco's modified Eagle's medium (DMEM, Sigma-Aldrich, St. Louis, MO, #D6046) supplemented with 10% fetal bovine serum (FBS, Sigma-Aldrich, #F4135) and 1% penicillin-streptomycin (PS) solution (Sigma-Aldrich, #P4333). U937 cells were cultured in RPMI-1640 medium (Sigma-Aldrich, #R8758) supplemented with 10% FBS and 1% PS. HMC3 cells were cultured in Eagle's minimum essential medium (EMEM, ATCC, #30-2003) containing 6 g/L glucose, 2 mM glutamine, 10% FBS, and 1% PS. Cells were placed in a humidified incubator containing 5% CO_2_ at 37°C.

### Collection of blood samples and lung tissues

Venous blood samples were collected from 24 non-small cell lung cancer (NSCLC) patients who were diagnosed as being in T0 stage and 24 ALI patients who were treated but died, and had signed organ donation consent when they were alive. All patients were therapied in the Department of Critical Care Medicine, Jiangxi Provincial People's Hospital, during 2009-2017. Blood samples were immediately centrifuged at 800 × *g* for 10 min to obtain serum, which was applied to measure the levels of cytokines including IL-1β (#ab214025), IL-4 (#ab215089), IL-6 (#ab100573), IL-13 (#ab46038), IL-15 (#ab100554), and TNF-α (ab181421) using ELISA kits purchased from Abcam (Cambridge, MA, USA). Normal lung tissue samples (noncancerous lung tissues) were collected from the 24 NSCLC patients described above when they underwent surgeries. The reason why we collected noncancerous lung tissue samples from NSCLC patients under T0 stage mainly included: (1) lung tissues of these patients only had small cancerous lesions but no obvious inflammation; (2) we can easily obtain noncancerous lung tissue samples when NSCLC patients were undergone surgeries to remove cancerous lesions. The 24 ALI lung tissues were immediately collected when patients died. Biopsies were cryopreserved in a -80°C ultralow freezer until use. All patients signed tissue collection consents that were reviewed and approved by the ethical board of the Jiangxi Provincial People's Hospital. The basic information of these patients is included in Supplementary Table-1.

### MiRNA isolation and GeneChip miRNA array

Three paired normal lung tissues and ALI lung tissues were subjected to miRNA isolation using an Ambion® PureLink® miRNA Isolation Kit (Thermo Fisher Scientific, Waltham, MA, #K157001) following the manufacturer's guidelines. A total of 0.5 μg of miRNA from each sample was applied to a GeneChip miRNA 4.0 array (Thermo Fisher Scientific, #902412) according to the manufacturer's instructions.

### Quantification of miRNAs by qRT-PCR

Quantification of miRNAs was performed as described previously 25. Accordingly, the expression levels of miR-7-5p, miR-21-5p, miR-95-5p, miR-182-5p, miR-199a-3p, and miR-448 were measured by qRT-PCR using TaqMan microRNA assays (Assay ID: 483061, 477975, 477935, 479221, 477961, and 478105, respectively) following the manufacturer's instructions. The relative miRNA expression levels were normalized using RNU6B as an internal control.

### Total RNA extraction and qRT-PCR detection

Total RNA from cells and tissues was extracted using a TRIzol^TM^ kit (Thermo Fisher Scientific, #15596026) following the manufacturer's protocol. After quantification using a NanoDrop 2000 instrument (Thermo Fisher Scientific, #ND-2000), 0.5 μg RNA of each sample was utilized to synthesize cDNA with a Verso cDNA Synthesis kit (Thermo Fisher Scientific, #AB1453A). The obtained cDNA was diluted 200-fold, followed by qRT‐PCR analyses using a SYBR Green Supermix kit (Bio-Rad, Hercules, CA, USA, #1725270). The primers for these assays are included in Supplementary Table-2. β-actin was set as an internal control to normalize individual gene expression according to the 2^-ΔCt^ method.

### Immunoblot analysis

Protein levels were measured by immunoblot analysis following a previous protocol 26. Tissues and cells were lysed in 1×RIPA lysis buffer (Abcam, #ab156034). After boiling with Laemmli SDS sample buffer (Sigma-Aldrich, #S3401), equal amount of total cell lysate of each sample was loaded onto 10% SDS-PAGE gels, followed by transfer to polyvinylidene difluoride (PVDF) membranes (Thermo Fisher Scientific, #LC2002). The immunoblots were then probed with primary antibodies including anti-HDAC1 (Abcam, #ab53091), anti-FOXP3 (Abcam, #ab450), anti-CtBP2 (BD Biosciences, San Jose, CA, USA, #612044), anti-NLRP1 (Biolegend, San Diego, CA, USA, #a679802), anti-Caspase-1 (Santa Cruz Biotechnology, Dallas, TX, USA, #sc-56036), and anti-GAPDH (Santa Cruz Biotechnology, #47724). Protein signals were recorded using enhanced chemiluminescence reagents (GE Healthcare Life Sciences, Piscataway, NJ, USA, #RPN2232).

### Immunoprecipitation (IP) and mass spectrometry analysis

The IP procedure was performed as described previously 27. Accordingly, cells expressing pCDNA3-2×Flag-FOXP3 or pCDNA3-2×Flag were lysed in 1×RIPA lysis buffer supplemented with a protease inhibitor cocktail (Thermo Fisher Scientific, #78430), followed by centrifuging at 13,000 rpm for 15 min. The supernatant was incubated with anti-Flag agarose beads (Sigma-Aldrich, #A2220) at 4°C for 2 h. After washing 5 times with 1×RIPA lysis buffer, the Flag-FOXP3-associated protein complex was separated in a 10% SDS-PAGE gel and then stained with Coomassie Brilliant Blue R 250 (Thermo Fisher Scientific, #20278) for 15 min. Protein bands were digested using a SMART Digest Trypsin Kit (Thermo Fisher Scientific, #60109101), followed by mass spectrometry analysis as described previously 27.

### Coimmunoprecipitation (Co-IP) analysis

The Co-IP procedure was performed as described previously 28. Briefly, different combinations of plasmids were cotransfected into cells, followed by incubation at 37°C for 48 h. The transfected cells were then lysed in 1×RIPA lysis buffer supplemented with a protease inhibitor cocktail. After centrifuging at 13,000 rpm for 15 min, the supernatant was incubated with anti-Flag agarose beads and anti-c-Myc agarose beads (Sigma-Aldrich, #A7470) at 4°C for 2 h. The beads were then washed 5 times with 1×RIPA lysis buffer, and protein interactions were determined using immunoblot assays.

### Chromatin immunoprecipitation (ChIP) assay

The ChIP assay procedure was performed as described previously 29, 30. In brief, cells were primarily washed with phosphate *buffered* saline (PBS) buffer, followed by crosslinking with 1% formaldehyde (Polysciences, Inc. Warrington, PA, USA, #18814) at 37°C for 15 min. The fixed cells were then subjected to a standard ChIP assay using a high-sensitivity ChIP kit (Abcam, #ab185913) following the manufacturer's instructions. The antibodies used in this assay included anti-HDAC1 (Abcam, #ab53091), anti-FOXP3 (Abcam, #ab450), and anti-CtBP2 (BD Biosciences, #612044). The enriched DNA was then subjected to qRT-PCR analyses using the primers listed in Supplementary Table-3.

### Statistical analysis

All experiments were independently performed at least three times. Data are presented as the mean ± SEM from a representative experiment. Statistical significance was set at a *P*<0.05 (*), *P* < 0.01 (**) and *P* < 0.001 (***) according to the results of the two-sided Student's *t* test.

## Results

### ALI was associated with elevated levels of pro-inflammatory cytokines

**Figure 1:**
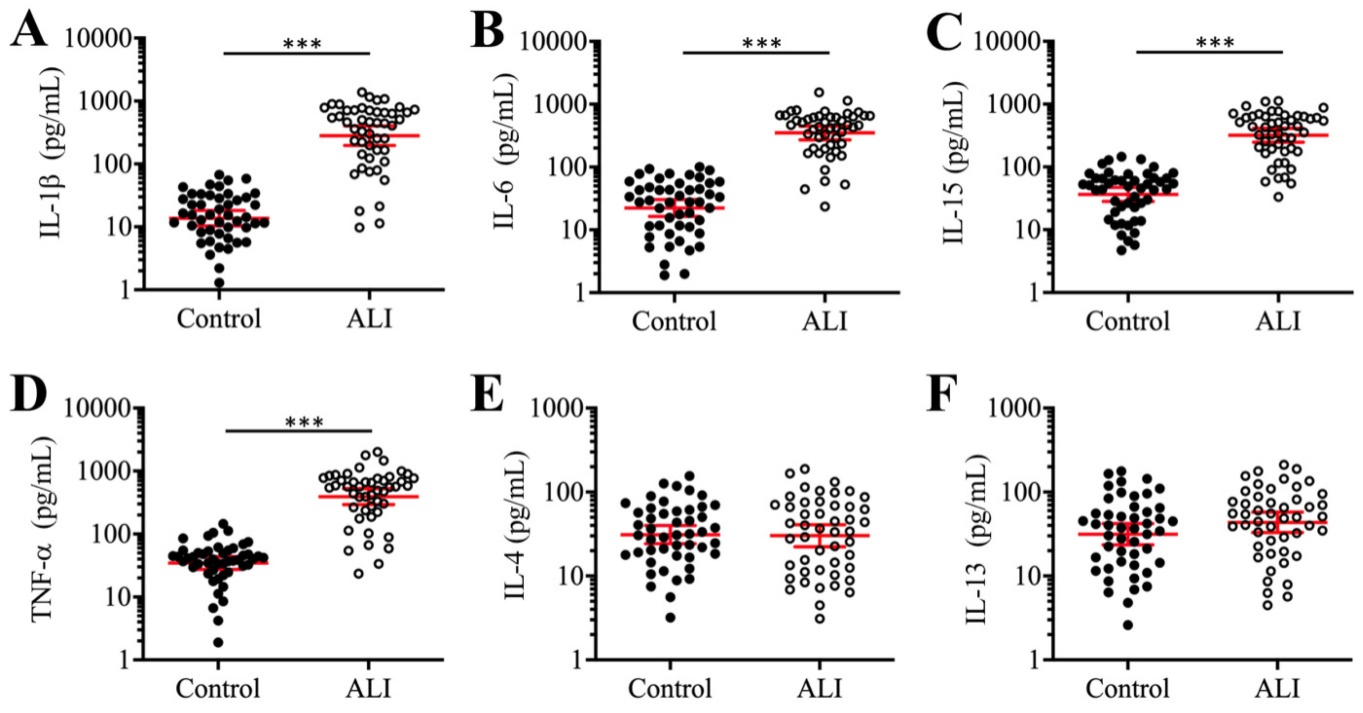
** The proinflammatory cytokines were elevated in ALI patients.** The concentrations of cytokines including IL-1β **(A)**, IL-6 **(B)**, IL-15 **(C)**, TNF-α **(D)**, IL-4 **(E)**, and IL-13 **(F)** were measured using ELISA kits in serum samples obtained from 24 NSCLC patients (Control) under T0 stage and 24 ALI patients. ***P < 0.001.

Previous studies have shown that the occurrence of ALI is associated with elevated levels of pro-inflammatory cytokines such as IL-1β, IL-6, IL-15, and TNF-α 9. To evaluate whether the levels of these cytokines were also increased in our ALI patients, we measured their concentrations in serum using ELISA. Our results indicated that the average concentrations of IL-1β, IL-6, IL-15, and TNF-α were significantly increased in the serum of 24 ALI patients compared to 24 non-ALI controls (Figures 1A-1D). The individual concentrations of these cytokines in the controls and ALI patients were as follows: for IL-1β, 14.3 pg/mL (range 1.94-98.35) versus 421.44 pg/mL (range 12.67-1865.33, *P* < 0.001; Figure 1A); for IL-6, 23.14 pg/mL (range 2.14-112.33) versus 342.76 pg/mL (range 31.34-1783.47, *P* < 0.001; Figure 1B); for IL-15, 33.62 pg/mL (range 5.32-165.88) versus 309.36 pg/mL (range 45.33-1157.85, *P* < 0.001; Figure 1C); and for TNF-α, 45.39 pg/mL (range 1.68-136.57) versus 396.43 pg/mL (range 23.74-2315.63, *P* < 0.001; Figure 1D). Moreover, we also measured the concentrations of two anti-inflammatory cytokines (IL-4 and IL-13) in the same serum samples. The results showed that both IL-4 and IL-13 levels were not significantly changed in the ALI patients compared to controls (Figures 1E and 1F). These results indicated that ALI patients exhibited significantly elevated pro-inflammatory cytokine levels and suggested that inflammation might play a dominant role in the pathogenesis of ALI.

### MiR-199a-3p was dramatically downregulated in ALI biopsies

**Figure 2:**
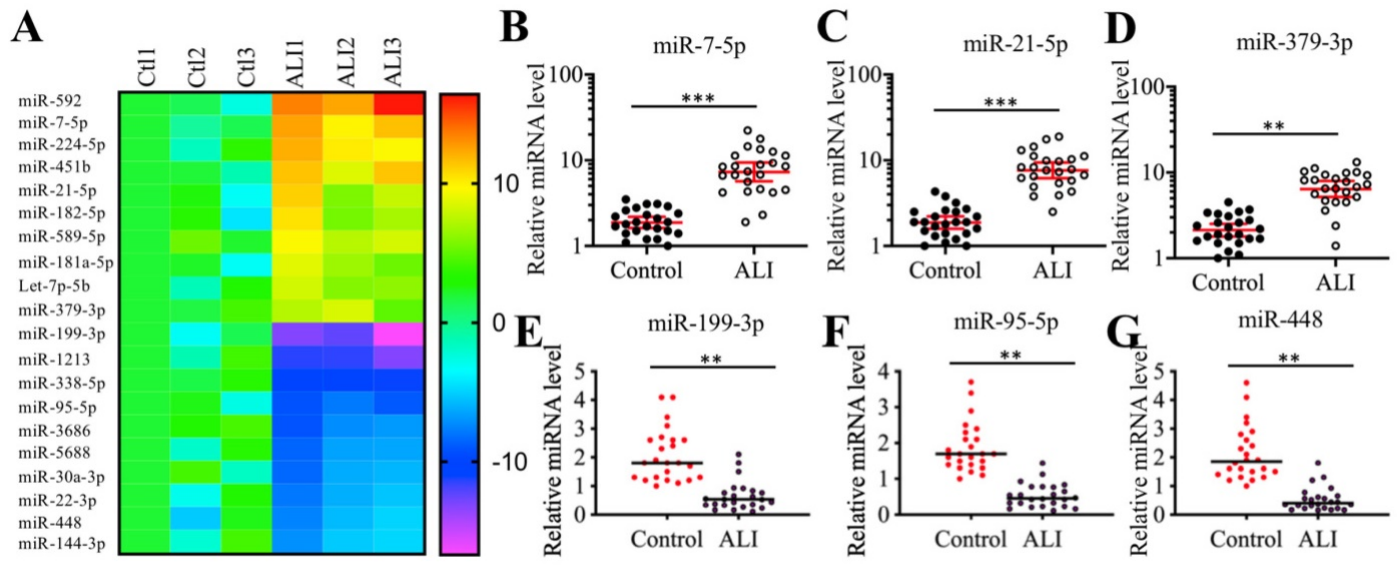
** Identification of aberrantly expressed miRNAs in ALI biopsies. (A)** Heat maps of 10 downregulated and 10 upregulated miRNAs with most obvious changes in ALI biopsies. The lung tissues of three NSCLC patients (Ctl1, -2, and -3) under T0 stage and three ALI patients (ALI1, -2, and -3) were applied to miRNA isolation, followed by a microarray analysis. The top 20 aberrantly expressed miRNAs were shown. **(B-G)** Verification of miRNA expression levels. Three upregulated miRNAs including miR-7-5p **(B)**, miR-21-5p **(C)**, and miR-379-3p **(D)**, and three downregulated miRNAs including miR-199a-3p **(E)**, miR-95-5p **(F)**, and miR-448 **(G)** were selected to verify their expression in lung tissues from 24 NSCLC patients (Control) and 24 ALI patients. ** P < 0.01 and *** P < 0.01.

Many miRNAs have been shown to play important roles in mouse ALI models 18-24. To identify miRNAs that were involved in the pathogenesis of human ALI, we conducted a miRNA-based microarray analysis using three-paired lung tissues from ALI patients and NSCLC patients under T0 stage. Filtering by a fold change of > 3.0 or <-3.0, we identified 44 upregulated and 62 downregulated miRNAs that were consistently expressed in all three ALI samples (Supplementary Table-4). As presented in Figure 2A, we listed 10 miRNAs showing the most obvious upregulation and downregulation. To further examine the accuracy of the microarray results, we randomly selected three upregulated (miR-7-5p, miR-21-5p and miR-379-3p) and three downregulated (miR-95-5p, miR-199a-3p and miR-448) miRNAs to verify their expression levels in lung tissues from ALI patients and controls. Consistent with the microarray results, we also observed that the expression levels of miR-7-5p, miR-21-5p and miR-379-3p were significantly upregulated, while the expression levels of miR-95-5p, miR-199a-3p and miR-448 were dramatically downregulated in the lung tissues from ALI patients compared to the controls. Based on the finding that miR-199a-3p showed the most obvious change in ALI lung tissues, we speculated that it might play an important role in the pathogenesis of ALI. Thus, we will focus our study on investigating the downstream targets of miR-199a-3p and revealing its upstream regulatory mechanism.

### LPS treatment repressed miR-199a-3p expression

**Figure 3:**
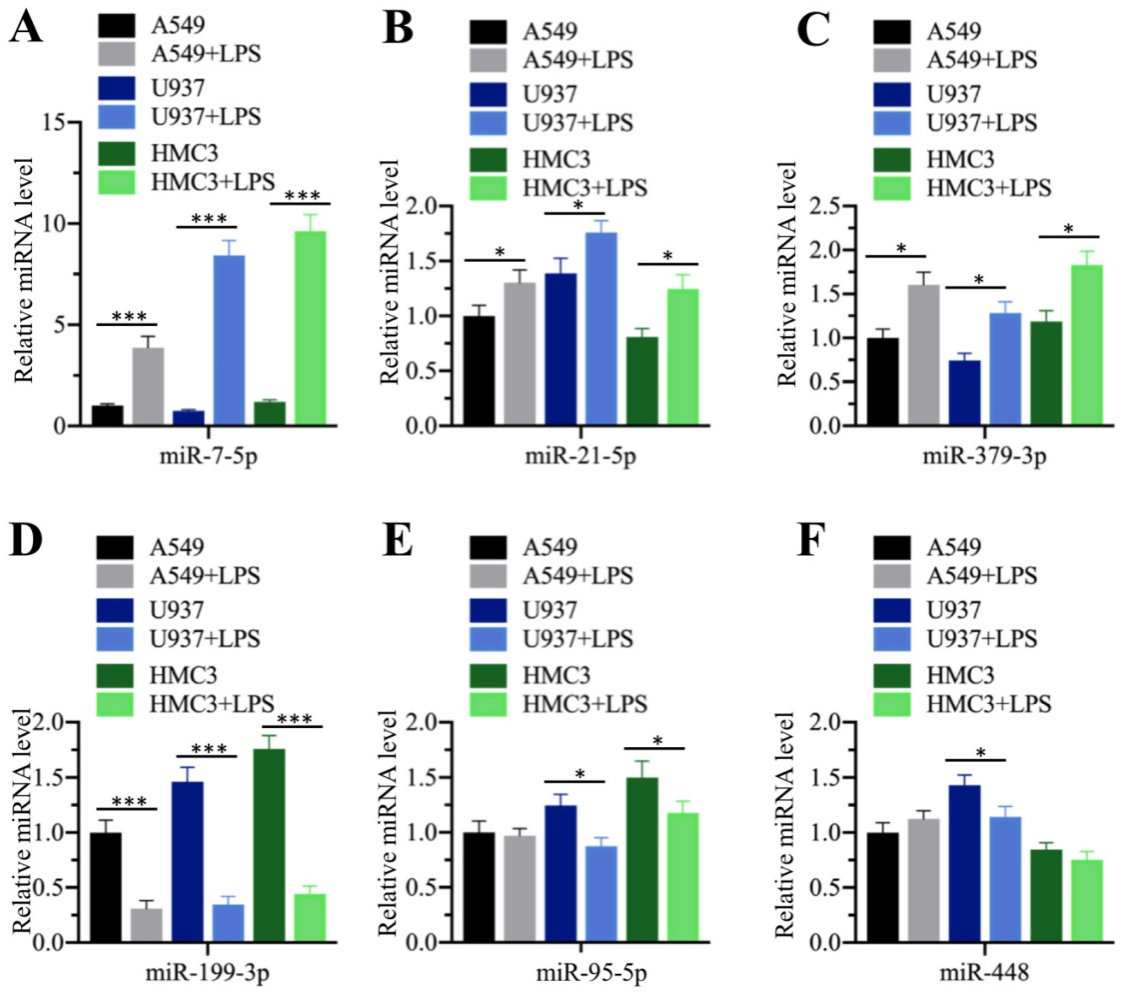
** LPS treatment repressed miR-199a-3p expression.** Three cell lines including A549, U937 and HMC3 were treated with 200 ng/mL LPS for 2 h, followed by measuring the expression levels of miR-7-5p **(A)**, miR-21-5p **(B)**, and miR-379-3p **(C)**, miR-199a-3p **(D)**, miR-95-5p **(E)**, and miR-448 **(F)**. * P < 0.05 and *** P < 0.01.

Our above results showed that the serum concentrations of pro-inflammatory cytokines were elevated in ALI patients, while miR-199a-3p was downregulated in ALI lung tissues. Their reverse expression levels encouraged us to determine whether inflammation status could affect miR-199a-3p level. For this purpose, we treated three different human cell lines, including A549 (epithelial), U937 (monocyte), and HMC3 (microglia), with LPS, which is one of the most powerful chemicals for inducing inflammatory response. After treatment, we measured the expression of the three upregulated (miR-7-5p, miR-21-5p and miR-379-3p) and three downregulated (miR-95-5p, miR-199a-3p and miR-448) miRNAs. Our results indicated that LPS affected the expression of these miRNAs to varying degrees (Figure 3). LPS significantly induced the upregulation of miR-7-5p (~4.2-10-fold) in all three cell lines (Figure 3A), while it only slightly increased the expression of miR-21-5p (~1.3-1.5-fold) and miR-379-3p (~1.5-1.8-fold) (Figures 3B and 3C). On the other hand, LPS dramatically repressed the expression of miR-199a-3p (~3.1-4.3-fold) (Figure 3D), while it only slightly inhibited the expression of miR-95-5p in U937 (~1.5-fold) and HMC3 (~1.5-fold) cells (Figure 3E) and that of miR-448 in U937 cells (~1.4-fold) (Figure 3F). These results clearly suggested that inflammation status could significantly repress miR-199a-3p expression.

### *NLRP1* was upregulated in ALI tissues and it was a target of miR-199a-3p

**Figure 4:**
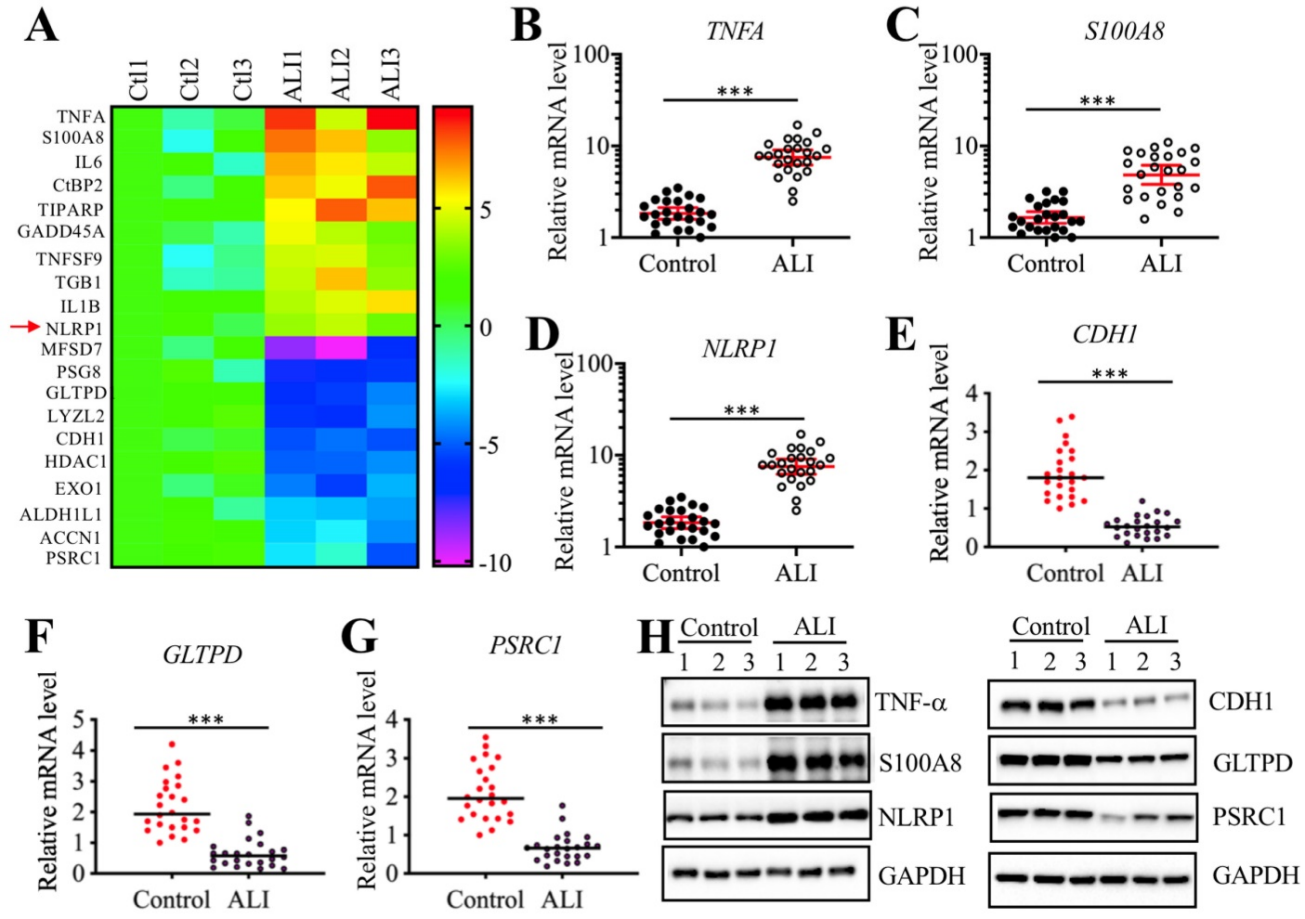
** Identification of aberrantly expressed genes in ALI biopsies. (A)** Heat maps of 10 downregulated and 10 upregulated genes with most obvious changes in ALI patients. The lung tissues of three NSCLC patients (Ctl1, -2, and -3) under T0 stage and three ALI patients (ALI1, -2, and -3) were applied to RNA isolation, followed by a microarray analysis. The top 20 aberrantly expressed genes were shown. **(B-G)** Verification of gene expression levels. Three upregulated genes including *TNFA* **(B)**, *S100A8* **(C)**, and *NLRP1* **(D)**, and three downregulated genes including *CDH1* **(E)**, *GLTPD* **(F)**, and *PSRC1* **(G)** were selected to verify their expression in 24 NSCLC patients and 24 ALI patients. *** P < 0.01. **(H)** Protein levels of TNF-α, S100A8 and NLRP1 in ALI lung tissues. The same tissue samples as microarray analysis were applied to western blotting analysis to examine protein levels of TNF-α, S100A8 and NLRP1. **(I)** Protein levels of CDH1, GLTPD and PSRC1 in ALI lung tissues. The same cell lysates used in (H) were subjected to immunoblots to examine protein levels of CDH1, GLTPD and PSRC1. GAPDH was used a loading control.

We next sought to identify the direct targets of miR-199a-3p. Target prediction was conducted in a database (www.mirdb.org) and we totally identified 477 potential targets of miR-199a-3p (Supplementary Table-5). Thus, it was difficult to determine which gene was the direct target of miR-199a-3p using this method. Based on the notion that the expression of miRNAs was reverse to their targets, we next sought to identify the miR-199a-3p targets by a microarray analysis by which we could obtain transcriptome profiles of ALI lung tissues. Using the same tissues employed for the miRNA-based microarray analysis, we identified 89 upregulated and 55 downregulated genes that were consistently expressed in all three ALI samples (Supplementary Table-6). As presented in Figure 4A, we listed 10 genes with the most obvious upregulation and downregulation. Interestingly, we found that some of these most obviously changed genes (e.g., *TNFA*, *IL-6*, and *NLRP1*) were involved in inflammation (Figure 4A). To further examine the accuracy of the microarray results, we selected three upregulated (*TNFA*, *S100A8*, and *NLRP1*) and three downregulated (*CDH1*, *GLTPD* and *PSRC1*) genes to verify their expression levels in lung tissues from ALI patients and controls. Consistently, the results showed that the expression levels of *TNFA*, *S100A8*, and *NLRP1* were significantly upregulated (Figures 4B-4D), while the expression levels of *CDH1*, *GLTPD* and *PSRC1* were dramatically downregulated in lung tissues from ALI patients compared to those from controls (Figures 4E-4G). We also detected their corresponding protein levels in the same tissues used in the microarray analysis. Consistent with their mRNA levels, the western blotting results indicated that the protein levels of TNF-α, S100A8, and NLRP1 were induced, while the protein levels of CDH1, GLTPD and PSRC1 were downregulated (Figures 4H and 4I). Comparing the 477 predicted targets of miR-199a-3p and 144 genes identified by microarray analysis, we found a shared gene known as *NLRP1*. More importantly, *NLRP1* was significantly upregulated in ALI biopsies (Figure 4D), which strongly suggested that *NLRP1* might be a direct target of miR-199a-3p.

**Figure 5:**
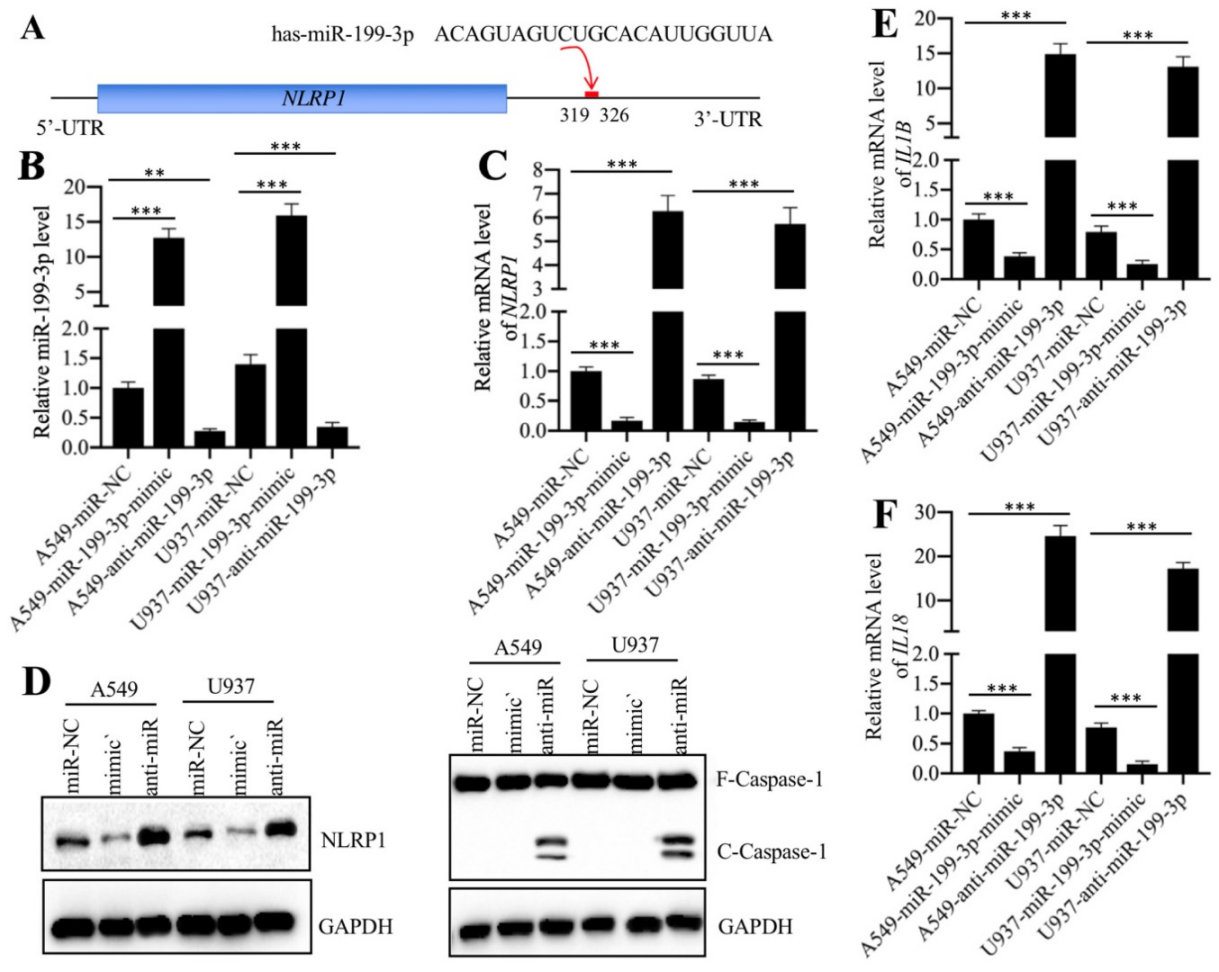
** NLRP1 was a target of miR-199a-3p. (A)** The 3′-UTR of *NLRP1* had a putative miR-199a-3p binding site. The potential binding position (319-326 bp after TGA) was shown with a red line. **(B)** The relative miR-199a-3p level in cells expressing miR-199a-3p-mimic and anti-miR-199a-3p. The A549 and U937 cells were transfected with miR-NC, miR-199a-3p-mimic, or anti-miR-199a-3p, followed by measuring the expression of miR-199a-3p in these cells by qRT-PCR assay. ** P < 0.01 and *** P < 0.001. **(C)** The *NLRP1* mRNA level was dependent on miR-199a-3p expression. The same samples used in (B) were used to examine *NLRP1* mRNA level. *** P < 0.001. **(D)** The activation of NLRP1 and Caspase-1 was dependent on miR-199a-3p expression. Cells used in (B) were applied to measure the protein levels of NLRP1 and Caspase-1 by immunoblots. GAPDH was used as a loading control. mimic: miR-199a-mimic; anti-miR: anti-miR-199a-3p. **(E** and **F)** The *IL1B* and *IL18* mRNA levels were dependent on miR-199a-3p expression. The same samples used in (B) were used to examine *IL1B* **(E)** and *IL18* **(F)** mRNA levels. *** P < 0.001.

Normally, miRNAs can directly bind to their targets through seed sequences 16. To verify that *NLRP1* was a direct target of miR-199a-3p, we primarily analyzed the 3'-UTR sequence of *NLRP1* and the miR-199a-3p sequence and found that miR-199a-3p might bind to the 3'-UTR of *NLRP1* in the 319-326 region (Figure 5A). Then, we transfected a negative control miRNA (miR-NC), a miR-199a-3p-mimic or an anti-miR-199a-3p into both A549 and U937 cells. After verifying their successful transfection (Figure 5B), we examined whether change of miR-199a-3p expression could affect *NLRP1* level. As shown in Figure 5C, we found that overexpression of miR-199a-3p caused downregulation of *NLRP1*, while inhibition of miR-199a-3p resulted in upregulation of *NLRP1* level. These results clearly showed that miR-199a-3p negatively regulated *NLRP1* expression. Given that NLRP1 is a component of the inflammasome and that its activation can induce Caspase-1 to cleave pro-IL-1β and pro-IL-18, eventually increasing the production of IL-1β and IL-18, we next sought to determine the protein levels of Caspase-1 and the mRNA levels of *IL-1B* and *IL-18* in miR-199a-3p-overexpressing or downregulating cells. Our results indicated that repression of miR-199a-3p activated Caspase-1 (Figure 5D), and increased *IL-1B* and *IL-18* mRNA levels (Figures 5E and 5F). On the other hand, overexpression of miR-199a-3p resulted in a decrease of both NLRP1 and Caspase-1 protein levels (Figure 5D), as well as caused downregulation of *IL-1B* and *IL-18* mRNA levels (Figures 5E and 5F). Thus, we concluded that *NLRP1* was a direct target of miR-199a-3p, and the elevated levels of IL-1β and IL-18 in the serum of ALI patients might be resulted from the downregulation of miR-199a-3p.

### The transcription factor FOXP3 (forkhead box P3) regulated miR-199a-3p levels

**Figure 6:**
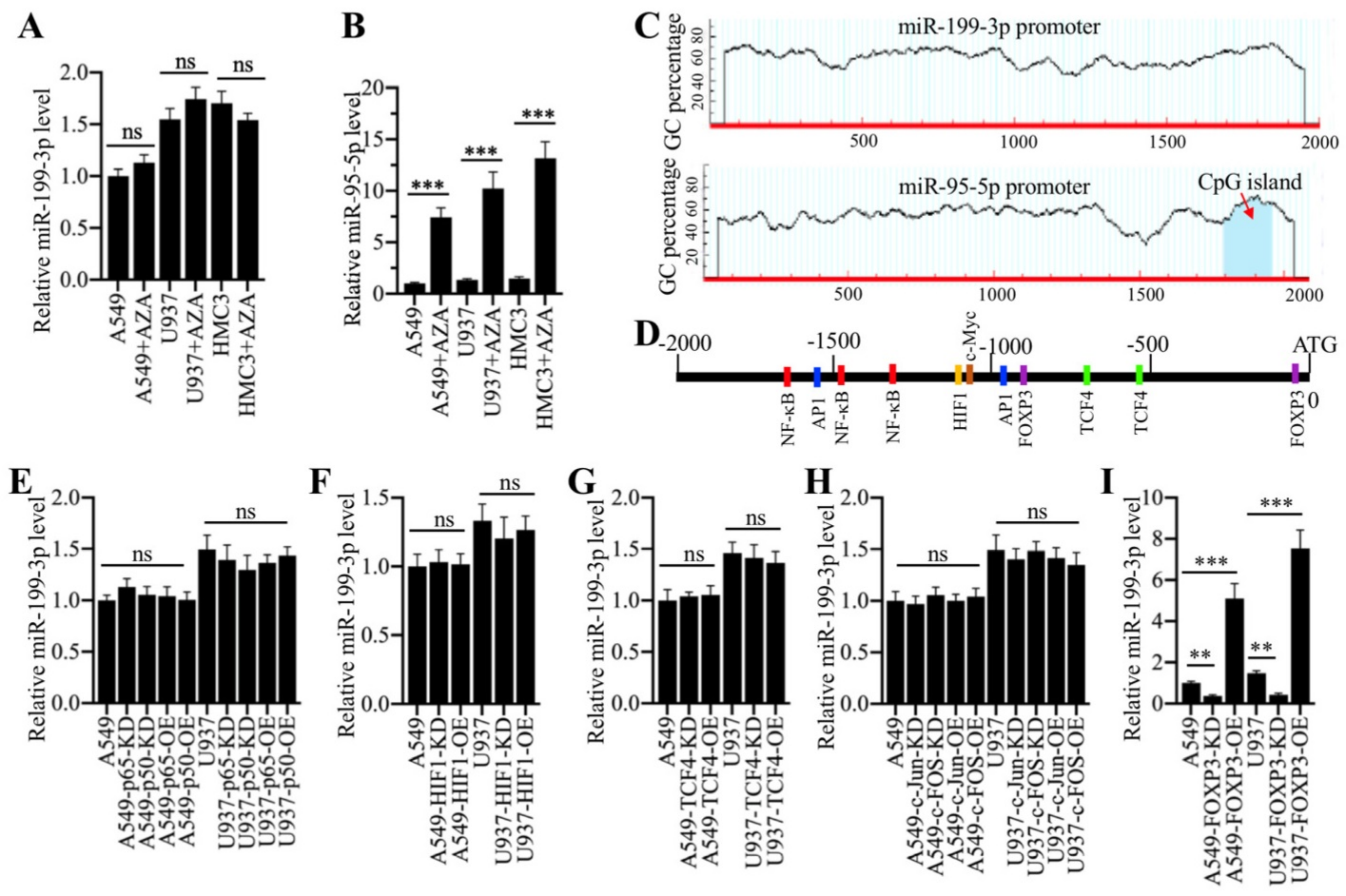
** Transcription factor FOXP3 regulated the expression of miR-199a-3p. (A)** DNA methylation inhibitor AZA treatment was not able to change miR-199a-3p level. Three cell lines including A549, U937 and HMC3 were treated with 50 μM AZA for 24 h, followed by examining miR-199a-3p level. ns represented no significance. **(B)** AZA treatment caused the significant upregulation of miR-95-5p. The same samples used in (A) were applied to measure miR-95-5p level. *** P < 0.001. **(C)** Identification of CpG island in the promoters of miR-199a-3p and miR-95-5p. A 2000 bp-length of miR-199a-3p or miR-95-5p promoter was applied to analyze the CpG island. CpG island was indicated by a red arrow. **(D)** A schematic diagram of transcription factor binding sites in the promoter of miR-199a-3p. The promoter of miR-199a-3p (2000 bp) was predicted the binding sites of different transcription factors. Five different transcription factors including NF-κB, AP-1, HIF1, TCF4 and FOXP3 were shown. **(E-I)** Effects of knockdown or overexpression of different transcription factors on the expression of miR-199a-3p. The A549 and U937 cells knocking down or overexpressing different transcription factors including *NF-κB* subunits **(E)**, *HIF1* **(F)**, *TCF4* **(G)**, *AP-1* subunits **(H)**, and *FOXP3* **(I)**, were subjected to RNA isolation, followed by qRT-PCR analyses to examine the expression of miR-199a-3p. ns represented no significant difference. **P < 0.01 and ***P < 0.001.

We next aimed to investigate the underlying mechanism of miR-199a-3p downregulation in ALI lung tissues. Given that DNA hypermethylation in the promoter region of a miRNA is a novel mechanism for its downregulation 16, 17, we primarily treated A549 and U937 cells with a DNA methylation inhibitor 5-Aza-2′-deoxycytidine (AZA) and then examined miR-199a-3p level. The results indicated that AZA treatment did not change miR-199a-3p level (Figure 6A). In contrast, AZA treatment significantly increased the expression of miR-95-5p (Figure 6B), whose promoter contained a CpG island (Figure 6C). One possible mechanism for miR-199a-3p downregulation was that it was controlled by transcription factors. To verify this hypothesis, we selected a 2000-bp length of the miR-199a-3p promoter and analyzed the potential transcription factors in this region in a database (www.alggen.lsi.upc.es). In total, we identified 11 potential transcription factor binding sites in this region, and these sites included three NF-κB (Nuclear factor kappa-light-chain-enhancer of activated B cells), two AP-1 (Activator protein 1), two TCF4 (transcription factor 4), two FOXP3, and one HIF1 (hypoxia-inducible factor 1) sites (Figure 6D). We next individually knocked down or overexpressed these transcription factors and subsequently determined whether their knockdown or overexpression affected miR-199a-3p level. Accordingly, we knocked down or overexpressed two NF-κB subunits (*p65* and *p50*), two AP1 subunits (*c-Jun* and *c-FOS*), *TCF4*, *HIF1* and *FOXP3* in A549 and U937 cells. After verifying their successful knockdown or overexpression (Supplementary Figure 1), we measured miR-199a-3p level in these cells. The results showed that knockdown or overexpression of *p65* and *p50* (Figure 6E), *HIF1* (Figure 6F), *TCF4* (Figure 6G), and *c-Jun* and *c-FOS* (Figure 6H) did not change miR-199a-3p level. However, knockdown or overexpression of *FOXP3* significantly downregulated or upregulated miR-199a-3p expression, respectively (Figure 6I). Moreover, we also examined the expression levels of *NLRP1*, *IL-1B* and *IL-18*. Our results showed that *FOXP3* downregulation or overexpression caused upregulation or downregulation of *NLRP1*, *IL-1B* and *IL-18*, respectively (Supplementary Figure 2), which suggested that FOXP3 could regulate the expression of miR-199a-3p and its downstream signaling.

### CHFTC repressed the expression of miR-199a-3p

**Figure 7:**
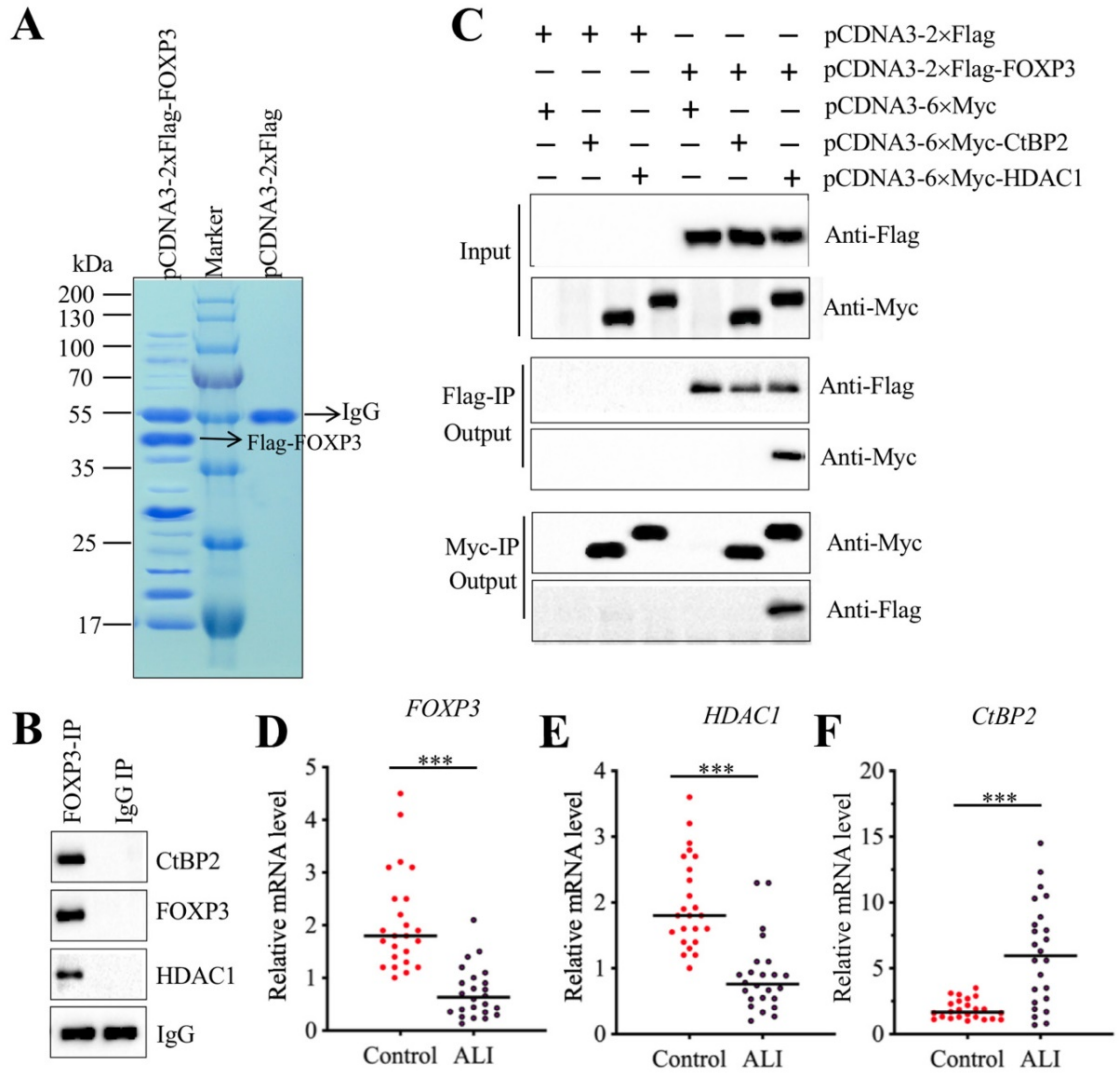
** FOXP3 formed a transcriptional complex with HDAC1 and CtBP2. (A)** The *in vivo* IP of the FOXP3-associated complex. A549 cells were transfected with pCDNA3-2×Flag and pCDNA3-2×Flag-FOXP3, respectively. After 48 h, cells were subjected to IP analysis with the anti-Flag agarose beads. After purification, the Flag-FOXP3-associated protein complex was separated by SDS-PAGE gel, followed by staining with Coomassie Blue. The IgG and Flag-FOXP3 bands were indicated by arrows. **(B)** FOXP3 associated with HDAC1 and CtBP2 in *vivo*. One ALI lung tissue was subjected to IP analysis with the anti-FOXP3 and anti-IgG antibodies. The purified protein complexes were subjected to examine the protein levels of CtBP2, FOXP3 and HDAC1. IgG was used as a loading control. **(C)** FOXP3 directly interacted with HDAC1 but not CtBP2. A549 cells were transfected with the following combinations of plasmids: pCDNA3-2×Flag + pCDNA3-6×Myc, pCDNA3-2×Flag + pCDNA3-6×Myc-CtBP2, pCDNA3-2×Flag + pCDNA3-6×Myc-HDAC1, pCDNA3-2×Flag-FOXP3 + pCDNA3-6×Myc, pCDNA3-2×Flag-FOXP3 + pCDNA3-6×Myc-CtBP2, and pCDNA3-2×Flag-FOXP3+ pCDNA3-6×Myc-HDAC1. After 48 h, cells were used for Co-IP analyses with anti-Myc-agarose or anti-Flag-agarose beads. **(D-F)** The mRNA levels of *FOXP3*, *HDAC1* and *CtBP2* in ALI biopsies. The qRT-PCR analyses were performed to examine the mRNA levels of *FOXP3* **(D)**, *HDAC1* **(E)** and *CtBP2* **(F)** in the lung tissues form 24 NSCLC and 24 ALI patients. ***P<0.001.

Transcription factors often interact with other proteins, such as histone modification enzymes and transcription corepressors or activators, to form a transcriptional complex that controls gene expression 31-33. To investigate the members of FOXP3-associated transcription complex, we constructed an overexpression vector pCDNA3-2×Flag-FOXP3 and transfected it into A549 cells. After IP purification, we obtained a Flag-FOXP3-associated protein complex (Figure 7A) and then applied it to a mass spectrometry analysis. In total, we obtained 78 potential candidates (Supplementary Table-7). After carefully studying the protein candidate lists, we identified a transcription corepressor CtBP2 (C-terminal-binding protein 2) and a histone deacetylase enzyme HDAC1. To determine whether FOXP3 could form a complex with CtBP2 and HDAC1, we performed an IP assay with anti-FOXP3 in an ALI lung tissue. After purification, we applied the FOXP3-associated complex to examine the existence of CtBP2 and HDAC1. As expected, we found that both CtBP2 and HDAC1 can be immunoprecipitated by FOXP3 (Figure 7B). Given that CtBP2 has been found to directly interact with HDAC1 previously 34, we next sought to determine the direct interactions of FOXP3-CtBP2 and FOXP3-HDAC1. Accordingly, we cotransfected different combinations of plasmids into A549 cells: pCDNA3-2×Flag + pCDNA3-6×Myc, pCDNA3-2×Flag + pCDNA3-6×Myc-CtBP2, pCDNA3-2×Flag + pCDNA3-6×Myc-HDAC1, pCDNA3-2×Flag-FOXP3 + pCDNA3-6×Myc, pCDNA3-2×Flag-FOXP3 + pCDNA3-6×Myc-CtBP2, and pCDNA3-2×Flag-FOXP3 + pCDNA3-6×Myc-HDAC1. *In vitro* Co-IP assays showed that FOXP3 can directly interact with HDAC1 but not CtBP2 (Figure 7C). Thus, we speculated that FOXP3 primarily bound to the promoter region of miR-199a-3p and then recruited HDAC1, which interacted with CtBP2, eventually forming CHFTC. Interestingly, we also found that the expression *FOXP3*, *HDAC1* and *CtBP2* was aberrant in ALI tissues according to the microarray results (Supplementary Table-6). To verify this phenomenon, we measured the mRNA levels of these genes in 24 ALI lung tissues. Our results showed that both *HDAC1* and *FOXP3* mRNA levels were dramatically downregulated in ALI lung tissues compared to those of controls (Figures 7D and 7E). In contrast, *CtBP2* mRNA level was significantly upregulated in the same samples (Figure 7F). These results imply that CtBP2 may play opposite roles to FOXP3 and HDAC1. Since our above results have shown that knockdown or overexpression of *FOXP3* significantly affected the expression of miR-199a-3p, *NLRP1*, *IL-1B* and *IL-18* (Figure 6I and Supplementary Figure 2), we next aimed to determine the effect of knockdown or overexpression of *CtBP2* on the expression of these genes. Contrary to the effect of *FOXP3*, our results showed that knockdown of *CtBP2* resulted in the overexpression of miR-199a-3p but downregulation of *NLRP1*, *IL-1B* and *IL-18*, while overexpression of* CtBP2* significantly downregulated miR-199a-3p level but upregulated *NLRP1*, *IL-1B* and *IL-18* mRNA levels (Supplementary Figure 3).

### CHFTC specifically bound to the promoter of miR-199a-3p

To further verify the different regulatory roles of CtBP2, FOXP3 and HDAC1, we examined FOXP3 and HDAC1 protein levels in *CtBP2*-overexpression and knockdown cells. Our results indicated that *CtBP2* knockdown caused an increase in both FOXP3 and HDAC1 levels (Supplementary Figure 4A), while *CtBP2* overexpression resulted in a decrease in both FOXP3 and HDAC1 levels (Supplementary Figure 4B). These results suggest that CtBP2 acted as a repressor to inhibit FOXP3-mediated transcription. We next sought to evaluate whether change of *CtBP2* level could affect the occupancy of FOXP3 and HDAC1 in the promoter of miR-199a-3p. Meanwhile, we also aimed to determine the binding position of FOXP3 in the promoter of miR-199a-3p because we identified two potential sites (Figure 6D). Accordingly, we performed ChIP assays in *CtBP2*-overexpression and knockdown cells. Our results indicated that the occupancy of FOXP3 and HDAC1 in the promoter of miR-199a-3p (-31 to -37 region instead of -960 to -966 region) was significantly increased in CtBP2-knockdown cells (Supplementary Figures 4C and 4D). In contrast, their occupancy was significantly decreased in CtBP2-overexpression cells (Supplementary Figures 4E and 4F). These results demonstrated that CHFTC could specifically bind to the promoter of miR-199a-3p through a conserved binding site (-31 to -37, CTAAACA).

Since LPS treatment resulted in the downregulation of miR-199a-3p, we speculated that it should also affect the expression of CHFTC members and the binding of this complex to the promoter of miR-199a-3p. To verify this hypothesis, we primarily determined the protein levels of CtBP2, HDAC1 and FOXP3 in A549 and U937 cells treated with LPS. As shown in Supplementary Figure 5A, LPS treatment slightly induced CtBP2 levels but significantly repressed HDAC1 and FOXP3 levels at the same time. We then performed ChIP assays in LPS-treated cells to evaluate the occupancy of CtBP2, HDAC1, and FOXP3 in the promoter of miR-199a-3p. The results indicated that LPS treatment significantly increased CtBP2 occupancy (Supplementary Figure 5B) but decreased the binding of HDAC1 and FOXP3 in the promoter of miR-199a-3p in comparison to untreated cells (Supplementary Figures 5C and 5D).

## Discussion

**Figure 8:**
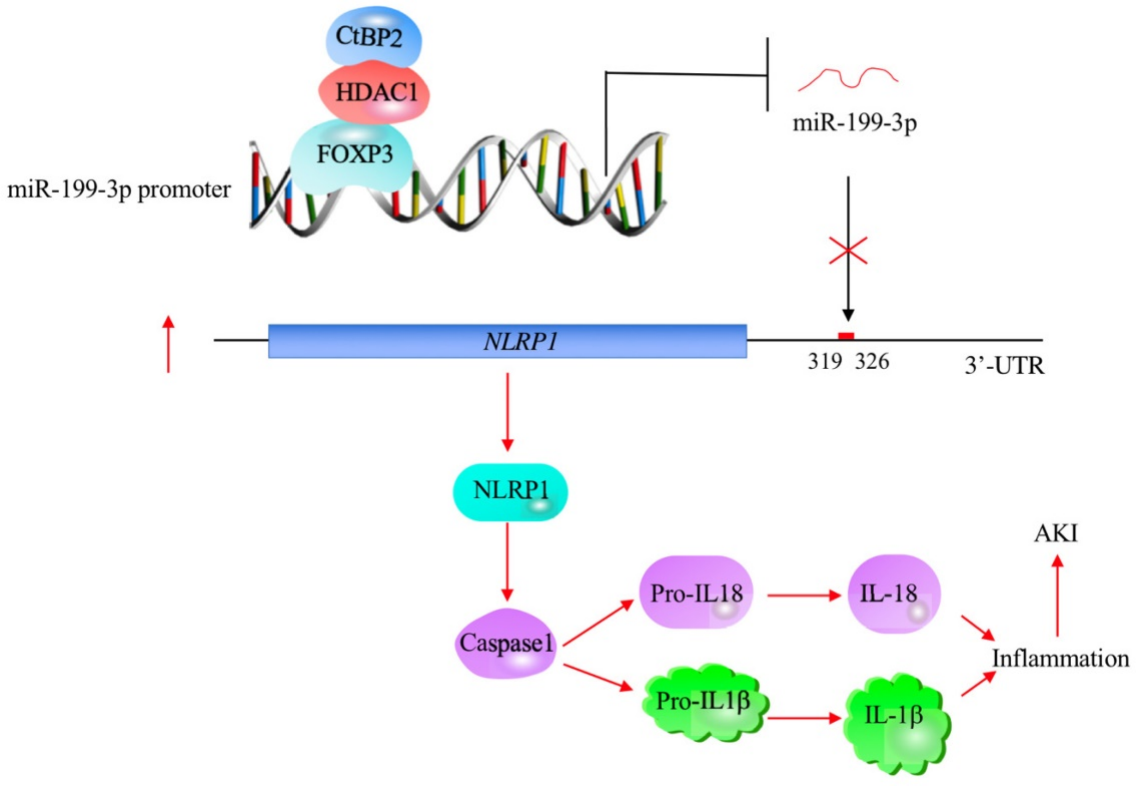
** A schematic model of CHFTC regulating miR-199a-3p expression and its downstream events in ALI.** CHFTC specifically binds to the promoter of miR-199a-3p. CtBP2 functions as a corepressor and its overexpression limits FOXP3 function and causes the downregulation of miR-199a-3p. The decrease of miR-199a-3p alleviates its inhibition against *NLRP1*, resulting the induction of NLRP1. The induced NLRP1 activates Caspase-1, which cleaves pro-IL-1β and pro-IL-18 to release the mature IL-1β and IL-18. The increased IL-1β and IL-18 levels exacerbate inflammatory response and cause the pathogenesis of ALI.

The profiling of miRNA expression provides extremely valuable information for investigating the pathological mechanisms of diseases 35. Similar to many other diseases, researchers have identified multiple miRNAs that are aberrantly expressed in ALI mouse models 18-24. However, their downstream targets and the mechanisms of their aberrant expression remain obscure 18-24. In this study, we used human ALI lung tissues to perform a microarray analysis and found that miR-199a-3p was extremely downregulated. We then focused our investigation on revealing the upstream and downstream signaling of miR-199a-3p. Our results support a model in which CHFTC specifically binds to the promoter of miR-199a-3p and negatively regulates its expression. The downregulation of miR-199a-3p releases its repression against *NLRP1*, causing an increase in *NLRP1* levels. The induced NLRP1 assembles into an inflammasome, which activates Caspase-1 to cleave pro-IL-1β and pro-IL-18. The release of mature IL-1β and IL-18 aggravates the inflammatory response, eventually leading to the occurrence of ALI (Figure 8).

Inflammation is a leading cause of ALI pathogenesis 1. However, limited information is available regarding the molecular changes that occur in this process. In the present study, we attempted to identify aberrantly expressed miRNAs to advance the understanding of these changes. Owing to the exclusive lung tissue samples from ALI patients, we found over 100 aberrantly expressed miRNAs, but we only focused our study on the miRNA with the most obvious change. In addition to miR-199a-3p, we found that other miRNAs have the potential to target genes involved in inflammation. For instance, *IL-1B* might be a target of miR-5688 (Supplementary Table-8). MiR-95-5p was predicted to bind to the 3'-UTR of *TGFB1* (Supplementary Table-8). We are currently determining whether these miRNAs also contribute to the pathogenesis of ALI by targeting genes involved in inflammation. In addition, the identified miRNA and gene expression profiles will greatly enhance our understanding of ALI pathogenesis.

In recent years, miR-199a-3p has been found to play multiple roles in different diseases by targeting many genes involved in different biological processes 36-39. For instance, the downregulation of miR-199a contributes to paclitaxel (PTX) resistance through targeting the oncogene Yamaguchi sarcoma viral homolog 1 (*YES1*) in prostate cancer 36. MiR-199a/b-3p inhibits gastric cancer cell proliferation via downregulating the PAK4 (p21 activated kinase 4)/MEK (mitogen-activated protein kinase kinase)/MAPK (mitogen-activated protein kinase 1) signaling pathway 37. MiR-199a-3p increases the cisplatin sensitivity of ovarian cancer cells by downregulating *ITGB8* (Integrin subunit Beta 8) expression 38. MiR-199a is downregulated in patients after coronary artery bypass graft surgery and is associated with increased *SIRT1* (Sirtuin 1) level 39. These findings clearly suggest that miR-199a-3p plays important roles in different biological processes through regulating the expression of multiple genes. However, none of these studies investigated the underlying mechanism of miR-199a-3p aberrant expression. To demonstrate the mechanism of miR-199a-3p downregulation in ALI lung tissues, we excluded the possibility of DNA methylation by treatment with a DNA methylation inhibitor AZA. Finally, we identified a transcriptional mechanism mediated by CHFTC.

To our knowledge, only a few studies have shown that transcription factors can mediate miRNA expression 40, 41. Fendler and colleagues found that the transcription factor HNF-1β (Hepatocyte Nuclear Factor 1-Beta) can regulate the expression of some circulating miRNAs, including miR-24, miR-223, miR-27b and miR-199a, in patients with diabetes 40. Mitxelena and colleagues found that the transcription factor E2F7 can regulate the transcription and maturation of multiple miRNAs, including miR-25, miR-26a, miR-27b and miR-92a, to restrain cell proliferation 41. In addition to the transcription factor-controlled regulation mechanism, the current understanding of aberrant miRNA expression mechanisms mainly includes DNA methylation and stimulation by exogenous and endogenous factors (e.g., LPS, steroid hormones and stresses), thereby affecting host genes 42. Our studies clearly showed that LPS can regulate the occupancy of CHFTC members in the promoter of miR-199a-3p, which implied that the inflammatory status of ALI patients might be the original contributor of miR-199a-3p aberrant expression. Moreover, CtBP-associated transcriptional complexes can regulate the expression of many genes 43-45. For instance, the CtBP2-p300-Runx2 complex has recently been found to function in the pathogenesis of atrophic nonunion by downregulating a number of bone development and differentiation genes, including *OSC (*osteocalcin*)*, *ALPL (*alkaline phosphatase*)*, *COL1A1 (*collagen 1a1*)*, *IBSP (*integrin binding sialoprotein*)*, *SPP1* (osteopontin), and *MMP13 (*matrix metallopeptidase 13*) 43.* The CtBP1-p300-FOXO3a complex can specifically repress the expression of two downstream targets, *Bax* and *Bim*, but not other known CtBP1 targets such as *CDH1* (epithelial Cadherin), *PTEN* (Phosphatase and Tensin Homolog), and *CDKN1A* (Cyclin-Dependent Kinase Inhibitor 1A) in human osteosarcoma cells 44. In these biological processes, CtBPs function as transcriptional corepressors to negatively regulate the expression of downstream targets 45. In addition, some studies have revealed that CtBPs exhibit transcriptional activation abilities. For instance, CtBP2 can directly activate the expression of *Tiam1* (T-cell lymphoma invasion and metastasis 1 (Tiam1) in an NADH-dependent manner 46. CtBP1 can activate the expression of *MDR1* (Multidrug Resistance 1), causing induction of P-glycoprotein and leading to drug resistance 47. CtBP1 can form a transcriptional complex with LSD1 (Lysine Demethylase 1) and CoREST (REST Corepressor 1) and this complex binds to the promoter of *NeuroD1* to transactivate its expression in gastrointestinal endocrine cells 48. In current study, we also observed the upregulation of *CtBP1* in ALI lung tissues but not found it in FOXP3-associated complex (Supplementary Table-6 and -7). The human CtBP1 and CtBP2 share over 80% protein sequence identity, which encouraged us to determine if CtBP1 was also involved in the regulation of miR-199a-3p expression. For this purpose, we knocked down or overexpressed *CtBP1* in A549 cells (Supplementary Figure 6A). Compared with *CtBP2*, we found that knockdown or overexpression of *CtBP1* could not significantly change miR-199a-3p and *NLRP1* levels (Supplementary Figures 6B and 6C). These results indicated that CtBP2 had a unique role in regulating miR-199a-3p and its target expression. Based on the results that *CtBP2* expression is much higher than *CtBP1*, we speculate one possible mechanism is that CtBP2 preferentially forms a transcriptional complex with HDAC1 and FOXP3, thereby limiting the function of CtBP1 in this process. In addition, some small molecules (e.g., NSC95397 and 4-methylthio-2-oxobutyric acid-MTOB) are able to target CtBPs to disrupt the downstream events in which they are involved 45, we will evaluate the effects of these small molecules on improving the symptoms of ALI in a mouse model.

In conclusion, we discovered that the transcription factor FOXP3 can associate with HDAC1 and CtBP2 to form a CHFTC protein complex, which specifically binds to the promoter of miR-199a-3p and represses its expression. The downregulation of miR-199a-3p alleviates its inhibition against *NLRP1* and causes induction of *NLRP1* and activation of its downstream events. Our studies may provide an avenue for targeting CHFTC to improve inflammatory response in the therapy of ALI.

## Supplementary Material

Supplementary figures and tables.
