# Supplementary material for: Downregulation of miR-199a-3p mediated by the CtBP2-HDAC1-FOXP3 transcriptional complex contributes to acute lung injury by targeting NLRP1
Source: Int J Biol Sci. 2019 Sep 8;15(12):2627–40. doi: 10.7150/ijbs.37133 (PMC6854378; doi:10.7150/ijbs.37133)
Supplement: Supplementary file 1 — Supplementary figures and tables. [file ijbsv15p2627s1.pdf]

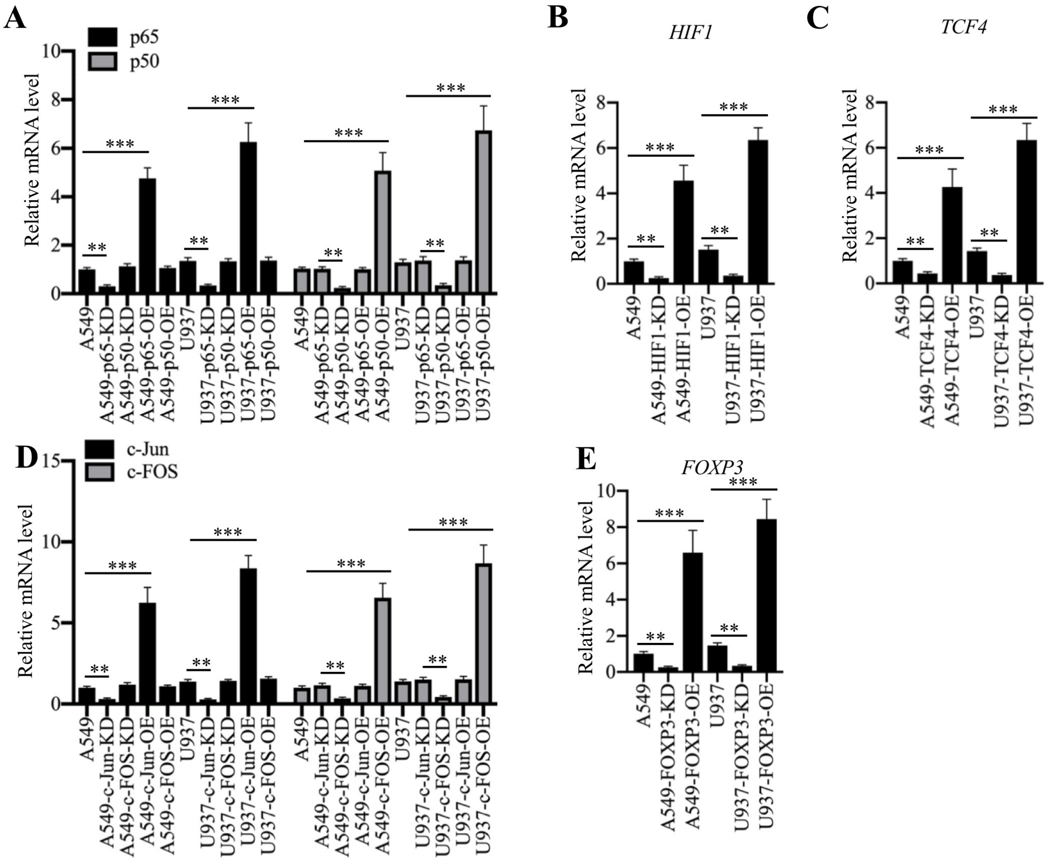

Supplementary Figure 1. qRT-PCR analyses for measuring the knockdown efficiency of transcription factors.

A549 and U937 cells knocking down or overexpressing different transcription factors including NF-κB subunits p65 and p50 **(A)**, HIF1 **(B)**, TCF4 **(C)**, AP-1 subunits c-Jun and c-FOS **(D)**, or FOXP3 **(E)** were subjected to RNA isolation, followed by qRT-PCR analyses to examine the expression of these transcription factors. $^{**}P<0.01$ and $^{***}P<0.001$.

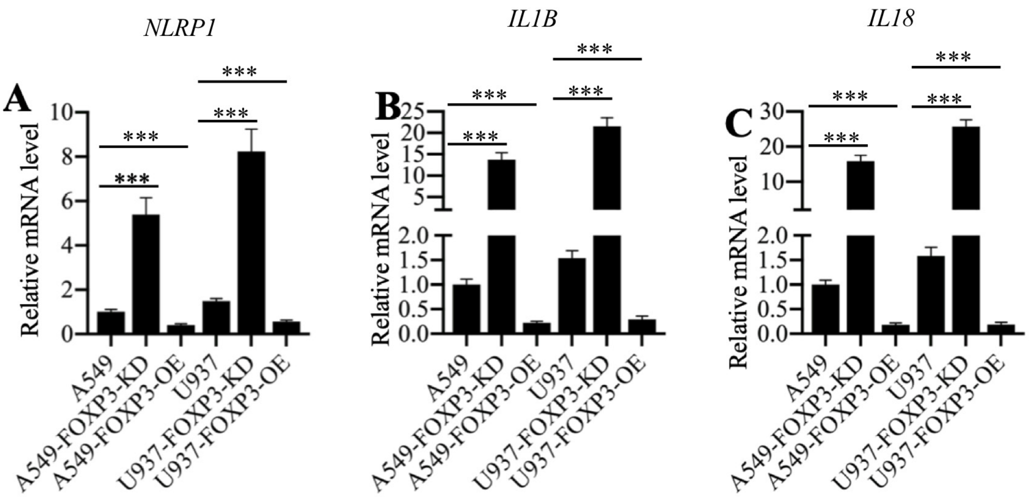

Supplementary Figure 2. Effects of *FOXP3* downregulation and overexpression on the expression of *NLRP1*, *IL1B* and *IL18*.

A549 and U937 cells were transfected with si-FOXP3 or pCDNA3-2×Flag-FOXP3. After 24 h, cells were subjected to RNA isolation, followed by qRT-PCR analyses to examine the expression of *NLRP1* **(A)**, *IL1B* **(B)** and *IL18* **(C)**. ****P*<0.001.

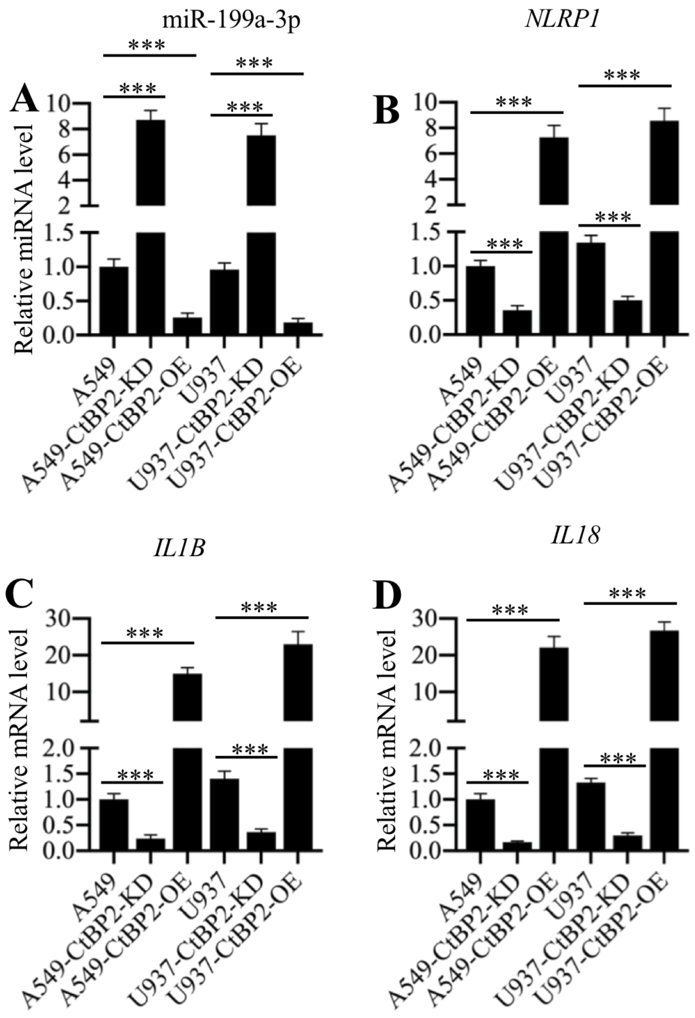

Supplementary Figure 3. Effects of *CtBP2* downregulation and overexpression on the expression of miR-199a-3p, *NLRP1*, *IL1B* and *IL18*.

A549 and U937 cells were transfected with si-CtBP2 or pCDNA3-2×Flag-CtBP2. After 24 h, cells were subjected to RNA isolation, followed by qRT-PCR analyses to examine the expression of miR-199a-3p **(A)**, *NLRP1* **(B)**, *IL1B* **(C)** and *IL18* **(D)**. ***P<0.001.

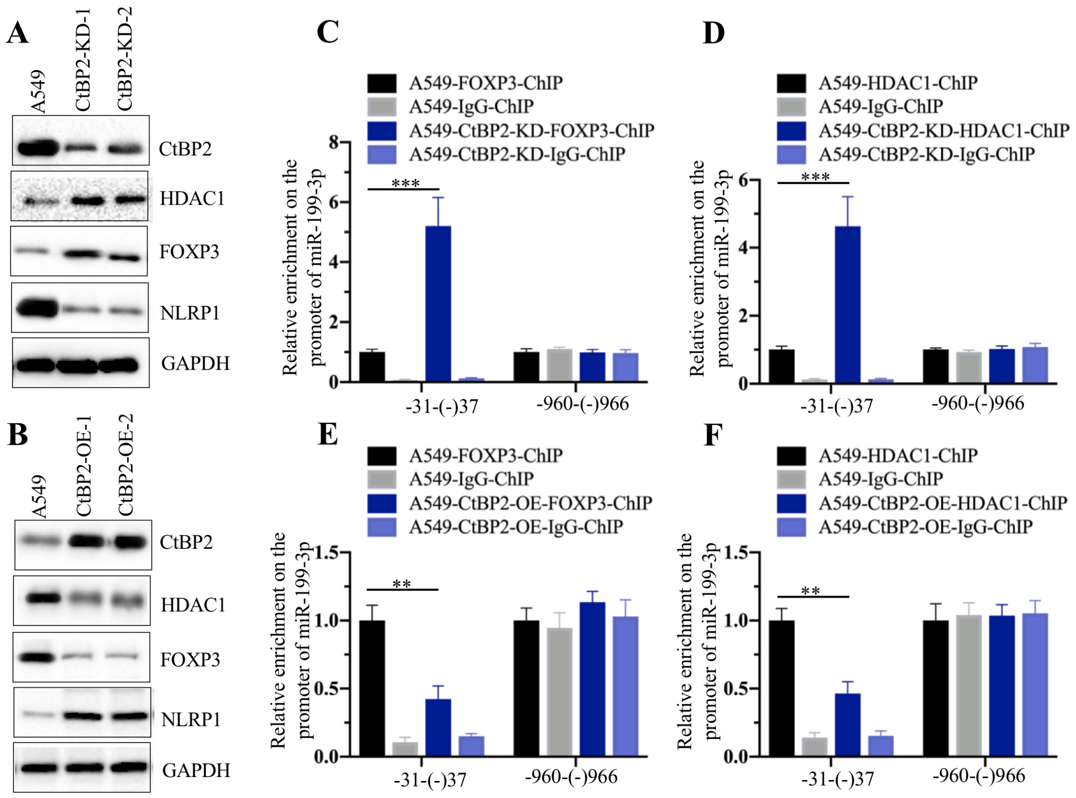

Supplementary Figure 4. CHFTC specifically bond to the promoter of miR-199a-3p.

(A and **B)** Effects of *CtBP2* knockdown and overexpression on HDAC1 and FOXP3 protein levels. A549 cells were transfected with two CtBP2-specific shRNAs and pCDNA3-2×Flag-CtBP2 to obtain two *CtBP2*-knockdown cell lines (KD-1 and KD-2) **(A)** and two *CtBP2*-overexpression cells lines (OE-1 and OE-2) **(B)**, respectively. These cells were used to examine the protein levels of CtBP2, HDAC1, FOXP3 and NLRP1. GAPDH was used as a loading control. **(C** and **D)** The enrichment of FOXP3 and HDAC1 in the promoters of miR-199a-3p was significantly increased upon *CtBP2* knockdown. The A549 and CtBP2-KD1 cells were subjected to ChIP assays with anti-FOXP3 **(C)**, anti-HDAC1 **(D)** and IgG antibodies. The purified DNA was

applied to qRT-PCR analyses to examine the enrichment of FOXP3 and HDAC1 in the promoter of miR-199a-3p. Primers used for qRT-PCR analyses located in the two regions [-31-(-)37 and -960-(-966)] of miR-199a-3p promoter containing the FOXP3 binding site. ***P<0.001. **(E** and **F)** The enrichment of FOXP3 and HDAC1 in the promoters of miR-199a-3p was dramatically decreased upon *CtBP2* overexpression. A549 and CtBP2-OE1 cells were subjected to ChIP assays with anti-FOXP3 **(E)**, anti-HDAC1 **(F)** and IgG antibodies. The purified DNA was applied to qRT-PCR analyses to examine the enrichment of FOXP3 and HDAC1 in the promoter of miR-199a-3p. **P<0.01.

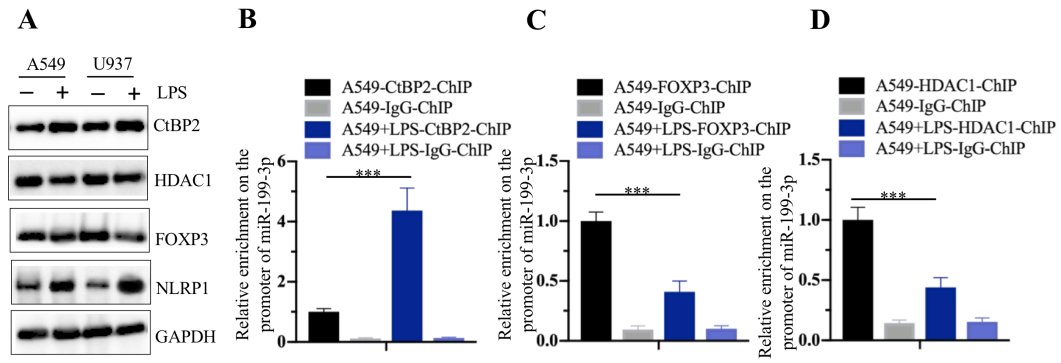

Supplementary Figure 5. LPS treatment changed the binding of CHFTC in the promoter of miR-199a-3p.

(A) LPS treatment increases CtBP2 protein levels. A549 and U937 cells were treated with 200 ng/mL LPS for 2 h, followed by protein isolation and immunoblot analysis to examine the protein levels of CtBP2, HDAC1, FOXP3 and NLRP1. GAPDH was used a loading control. **(B-D)** LPS treatment affected the enrichment of CtBP2, FOXP3 and HDAC1 in the promoter of miR-199a-3p. A549 and U937 cells were treated with 200 ng/mL LPS for 2 h, followed by ChIP assays with anti-CtBP2 **(B)**, anti-FOXP3 **(C)**, anti-HDAC1 **(D)** and IgG antibodies. The purified DNA was applied to qRT-PCR analyses to examine the enrichment of CtBP2, FOXP3 and HDAC1 in the promoters of miR-199a-3p. ***P<0.001.

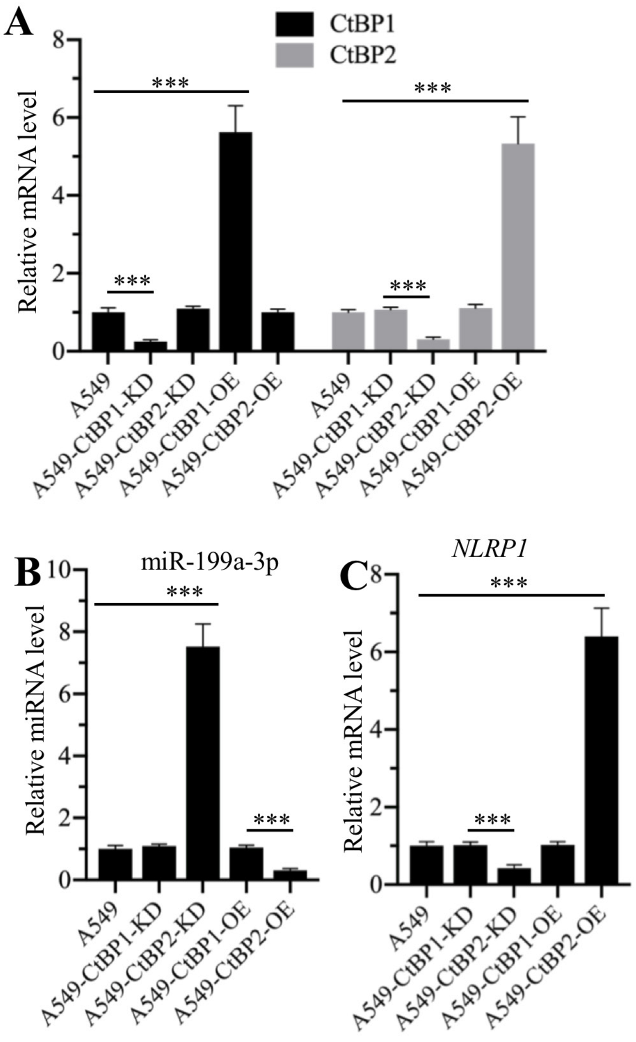

Supplementary Figure 6. Effects of *CtBP1* downregulation and overexpression on the expression of miR-199a-3p and *NLRP1*.

A549 cells were transfected with si-CtBP1, si-CtBP2, pCDNA3-2×Flag-CtBP1 and pCDNA3-2×Flag-CtBP2, respectively. After 24 h, cells were subjected to RNA isolation, followed by qRT-PCR analyses to examine the expression of *CtBPs* **(A)**, miR-199a-3p **(B)** and *NLRP1* **(C)**. ***P<0.001.

Supplementary Table-1. The basic information of NSCLC and ALI patients (n=24 in each group)

Parameter	NSCLC	ALI
Mean age	57.8±5.7	48.2±6.8
Gender	12M/12F	17M/7F
Stage	T0	Died

F, female; M, male.

Supplementary Table-2. Primers used for qRT-PCR analyses

Gene	Forward Primers	Reverse primers
TNFA	5’- GCAACAAGACCACCACTTCG-3’	5’- CTCAAGTCCTGCAGCATTC-3’
S100A8	5’- GCTAGAGACCGAGTGTCCTCA-3’	5’-CACGCCCATCTTTATCACCAGA-3’
NLRP1	5’- ATGCCCTGCGCTTCATTCCCG-3’	5’- CATTTCTAGATCATCTATGGC-3’
CDH1	5’- CACCACTGGGCTGGACCGAG-3’	5’-TGGGATTGAAGATCGGAGGA-3’
GLTPD	5’-TGTGAGCTGAGCTGGTTAG-3’	5’- GCACCCTGGCGAGGCCTGGCT-3’
PSRC1	5’- TGCAGGACACAGAGTGCG-3’	5’- TCCAGGTACTGACTGTAGTGG-3’
IL1B	5’- TGTACCTGTCCTGCGTGTT-3’	5’- CTCCCAGGAAGACGGGCATG-3’
IL18	5’-CCAGCCTAGAGGTATGGCT-3’	5’- TATCCCCCAATTCATCCTC-3’
IL-6	5’-CAACCTGAACCTTCCAAAGATG-3’	5’- ACTCATCTGCACAGCTCTGG-3’
p65	5’-AGTGCGGGACCCATCAGGCA-3’	5’- TCATCCCCACCGAGGCAGCT-3’
P50	5’-AGCTAATCCGCCAAGCAGCT-3’	5’- TTTCAAGTTGGATGCATTGG-3’
HIF1	5’- GAGCTTGCTCATCAGTTG-3’	5’- TGTCATCTTCAATATCC-3’
TCF4	5’-TGCTCCATCAGCAAGCACT-3’	5’- TCCTGCATAGCCAGGCTG-3’
c-Jun	5’-GCCTTCGTTAACTGTGTAT-3’	5’- AACACTGGGCAGGATACCC-3’
c-FOS	5’-ACTCCAGGGCTGGCGTT-3’	5’-CTTGGAGTGTATCAGTCAGC-3’
FOXP3	5’-CAAAGCCTCAGACCTGCTGG-3’	5’-AGGGTGCCACCATGACTAG-3’
□-Actin	5’-AGAGCTACGAGCTGCCTGAC-3	5’- AGCACTGTGTTGGCGTACAG -3’

Supplementary Table-3. Primers used for ChIP qRT-PCR analyses

Gene	Forward Primers	Reverse primers
miR-199a-3p promoter (-31-37)	5'- AGCTCTCCAGTGGCGGCG-3'	5'- CAGAGGACACCTCCACTCCG-3'
miR-199a-3p promoter (-960-966)	5'- TGATCTCATGCCTGAGCC-3'	5'- CACACACACACACAAACACA-3'

Supplementary Table-4. The aberrant expressed microRNAs in ALI lung tissues

miRNA	Average fold change	P Value	Expression
miR-592	22.5	0.0045	Up
miR-7-5p	21.6	0.00093	Up
miR-224-5p	21.2	0.0025	Up
miR-451b	20.4	0.0052	Up
miR-21-5p	19.7	0.00081	Up
miR-182-5p	19.6	0.0033	Up
miR-589-5p	19.2	0.00062	Up
miR-181a-5p	18.8	0.0045	Up
Let-7p-5b	17.9	0.0026	Up
miR-379-3p	17.5	0.0056	Up
miR-18a	17.1	0.012	Up
miR-32	16.6	0.0053	Up
miR-100	16.2	0.0067	Up
miR-328	15.4	0.0025	Up
miR-132	14.9	0.023	Up
miR-340	14.8	0.0034	Up
miR-485	14.6	0.0017	Up
miR-490	14.1	0.0066	Up
miR-892a	13.7	0.0038	Up
miR-943	13.4	0.0015	Up
miR-921	12.9	0.00042	Up
miR-1231	12.5	0.0015	Up
miR-1305	12.1	0.0091	Up
miR-17	11.7	0.00024	Up
miR-2682	11.4	0.00011	Up
miR-302c	10.9	0.00053	Up
miR-23a	10.7	0.00056	Up
miR-518d	10.2	0.00032	Up
miR-125a	9.9	0.0094	Up
miR-106a	9.8	0.00057	Up

miR-210	9.5	0.0083	Up
miR-370	9.1	0.00021	Up
miR-377	8.8	0.0052	Up
miR-429	8.5	0.0025	Up
miR-422a	7.9	0.0016	Up
miR-650	7.7	0.0057	Up
miR-933	7.1	0.0024	Up
miR-770	6.6	0.00093	Up
miR-1200	6.0	0.0033	Up
miR-1243	5.3	0.00042	Up
miR-607	5.1	0.0051	Up
miR-633	4.7	0.0098	Up
miR-520d	4.2	0.00065	Up
miR-323b	3.5	0.0033	Up
miR-199a-3p	-29.1	0.00013	Down
miR-1213	-28.4	0.0016	Down
miR-338-5p	-27.9	0.00043	Down
miR-95-5p	-27.2	0.0051	Down
miR-3686	-26.7	0.00064	Down
miR-5688	-26.3	0.0075	Down
miR-30a-3p	-25.9	0.0037	Down
miR-22-3p	-25.8	0.0054	Down
miR-448	-25.5	0.0077	Down
miR-144-3p	-25.1	0.0052	Down
miR-555	-24.6	0.025	Down
miR-569	-24.2	0.0031	Down
miR-300	-23.9	0.0029	Down
miR-10a	-23.7	0.0095	Down
miR-15b	-23.3	0.00044	Down
miR-126	-23.1	0.0013	Down
miR-140	-22.9	0.0077	Down
miR-185	-22.5	0.00056	Down

miR-222	-22.2	0.0016	Down
miR-211	-21.9	0.00022	Down
miR-410	-21.5	0.0065	Down
miR-600	-21.2	0.0036	Down
miR-2113	-20.9	0.00024	Down
miR-762	-20.5	0.00016	Down
miR-631	-20.2	0.0011	Down
miR-770	-20.0	0.00076	Down
miR-1227	-19.6	0.0032	Down
miR-889	-18.9	0.00054	Down
miR-875	-18.3	0.0011	Down
miR-920	-17.3	0.0012	Down
miR-424	-17.0	0.0032	Down
miR-335	-16.9	0.00045	Down
miR-449c	-16.7	0.0032	Down
miR-509-1	-15.9	0.0055	Down
miR-1306	-15.2	0.00014	Down
miR-1537	-14.7	0.00065	Down
miR-653	-14.1	0.0012	Down
miR-676	-13.4	0.0016	Down
miR-3122	-12.9	0.00047	Down
miR-1294	-12.4	0.00068	Down
miR-8086	-11.9	0.00052	Down
miR-935	-11.6	0.00081	Down
miR-607	-11.1	0.00054	Down
miR-887-5p	-10.5	0.00091	Down
miR-135b-5p	-10.1	0.00043	Down
miR-619-5p	-9.5	0.00022	Down
miR-744-3p	-9.2	0.0094	Down
miR-1305	-8.8	0.0035	Down
miR-1290	-8.5	0.0043	Down
miR-559	-7.9	0.0035	Down

miR-876-5p	-7.4	0.0036	Down
miR-759	-6.9	0.0073	Down
miR-4324	-6.7	0.0046	Down
miR-4504	-5.9	0.00032	Down
miR-552-3p	-5.5	0.00075	Down
miR-466	-5.2	0.00084	Down
miR-363-3p	-4.9	0.00024	Down
miR-606	-4.5	0.00015	Down
miR-612	-4.2	0.0072	Down
miR-940	-3.9	0.00013	Down
miR-874-5p	-3.6	0.00066	Down
miR-2113	-3.1	0.00023	Down

Supplementary Table-5. The predicted targets of miR-199a-3p

miRDB

There are 477 predicted targets for hsa-miR-199a-3p in miRDB.

Target Detail	Target Rank	Target Score	miRNA Name	Gene Symbol	Gene Description
Details	1	100	hsa-miR-199a-3p	ADAMTSL3	ADAMTS like 3
Details	2	99	hsa-miR-199a-3p	KLHL3	kelch like family member 3
Details	3	99	hsa-miR-199a-3p	KATNBL1	katanin regulatory subunit B1 like 1
Details	4	99	hsa-miR-199a-3p	CELSR2	cadherin EGF LAG seven-pass G-type receptor 2
Details	5	99	hsa-miR-199a-3p	ITGA3	integrin subunit alpha 3
Details	6	99	hsa-miR-199a-3p	MAP3K4	mitogen-activated protein kinase kinase kinase 4
Details	7	98	hsa-miR-199a-3p	ETNK1	ethanolamine kinase 1
Details	8	98	hsa-miR-199a-3p	NID2	nidogen 2
Details	9	98	hsa-miR-199a-3p	NAA25	N(alpha)-acetyltransferase 25, NatB auxiliary subunit
Details	10	98	hsa-miR-199a-3p	LRP2	LDL receptor related protein 2
Details	11	97	hsa-miR-199a-3p	NOVA1	NOVA alternative splicing regulator 1
Details	12	97	hsa-miR-199a-3p	GNA12	G protein subunit alpha 12
Details	13	97	hsa-miR-199a-3p	SERPINE2	serpin family E member 2
Details	14	96	hsa-miR-199a-3p	PAWR	pro-apoptotic WT1 regulator
Details	15	96	hsa-miR-199a-3p	CD2AP	CD2 associated protein
Details	16	96	hsa-miR-199a-3p	BCAR3	BCAR3, NSP family adaptor protein
Details	17	96	hsa-miR-199a-3p	GALNT7	polypeptide N-acetylgalactosaminyltransferase 7
Details	18	96	hsa-miR-199a-3p	CDNF	cerebral dopamine neurotrophic factor
Details	19	96	hsa-miR-199a-3p	PAK4	p21 (RAC1) activated kinase 4
Details	20	96	hsa-miR-199a-3p	CEP85L	centrosomal protein 85 like
Details	21	95	hsa-miR-199a-3p	PHLPP2	PH domain and leucine rich repeat protein phosphatase 2
Details	22	95	hsa-miR-199a-3p	ITGB8	integrin subunit beta 8
Details	23	95	hsa-miR-199a-3p	PPP2R5E	protein phosphatase 2 regulatory subunit B'epsilon
Details	24	95	hsa-miR-199a-3p	MAP3K5	mitogen-activated protein kinase kinase kinase 5
Details	25	95	hsa-miR-199a-3p	ERBB4	erb-b2 receptor tyrosine kinase 4
Details	26	95	hsa-miR-199a-3p	TUBGCP3	tubulin gamma complex associated protein 3
Details	27	95	hsa-miR-199a-3p	SINHCAF	SIN3-HDAC complex associated factor
Details	28	95	hsa-miR-199a-3p	ASAP2	ArfGAP with SH3 domain, ankyrin repeat and PH domain 2
Details	29	94	hsa-miR-199a-3p	LIN28B	lin-28 homolog B
Details	30	94	hsa-miR-199a-3p	NLRP1	NLR family pyrin domain containing 1
Details	31	94	hsa-miR-199a-3p	RUNX1	runt related transcription factor 1
Details	32	94	hsa-miR-199a-3p	ATRX	ATRX, chromatin remodeler
Details	33	94	hsa-miR-199a-3p	APLP2	amyloid beta precursor like protein 2
Details	34	94	hsa-miR-199a-3p	SCD	stearoyl-CoA desaturase

Details	35	94	hsa-miR-199a-3p	G3BP2	G3BP stress granule assembly factor 2
Details	36	94	hsa-miR-199a-3p	SLC39A10	solute carrier family 39 member 10
Details	37	94	hsa-miR-199a-3p	ARHGEF3	Rho guanine nucleotide exchange factor 3
Details	38	94	hsa-miR-199a-3p	ZHX1	zinc fingers and homeoboxes 1
Details	39	94	hsa-miR-199a-3p	RAPH1	Ras association (RalGDS/AF-6) and pleckstrin homology domains 1
Details	40	94	hsa-miR-199a-3p	SEMA3A	semaphorin 3A
Details	41	94	hsa-miR-199a-3p	RFX3	regulatory factor X3
Details	42	94	hsa-miR-199a-3p	ADAM10	ADAM metallopeptidase domain 10
Details	43	93	hsa-miR-199a-3p	WFDC8	WAP four-disulfide core domain 8
Details	44	93	hsa-miR-199a-3p	ALX4	ALX homeobox 4
Details	45	93	hsa-miR-199a-3p	CDK7	cyclin dependent kinase 7
Details	46	93	hsa-miR-199a-3p	ACVR2A	activin A receptor type 2A
Details	47	93	hsa-miR-199a-3p	KIAA0319L	KIAA0319 like
Details	48	93	hsa-miR-199a-3p	QKI	QKI, KH domain containing RNA binding
Details	49	93	hsa-miR-199a-3p	RB1	RB transcriptional corepressor 1
Details	50	93	hsa-miR-199a-3p	NACC1	nucleus accumbens associated 1
Details	51	92	hsa-miR-199a-3p	PCDH7	protocadherin 7
Details	52	92	hsa-miR-199a-3p	COL12A1	collagen type XII alpha 1 chain
Details	53	92	hsa-miR-199a-3p	TPPP	tubulin polymerization promoting protein
Details	54	92	hsa-miR-199a-3p	PTPN3	protein tyrosine phosphatase, non-receptor type 3
Details	55	92	hsa-miR-199a-3p	EPG5	ectopic P-granules autophagy protein 5 homolog
Details	56	91	hsa-miR-199a-3p	SLC20A2	solute carrier family 20 member 2
Details	57	91	hsa-miR-199a-3p	KDM6A	lysine demethylase 6A
Details	58	91	hsa-miR-199a-3p	ADAMTS3	ADAM metallopeptidase with thrombospondin type 1 motif 3
Details	59	91	hsa-miR-199a-3p	DNMT3A	DNA methyltransferase 3 alpha
Details	60	91	hsa-miR-199a-3p	CYB5R4	cytochrome b5 reductase 4
Details	61	91	hsa-miR-199a-3p	DEPDC1B	DEP domain containing 1B
Details	62	91	hsa-miR-199a-3p	C9orf40	chromosome 9 open reading frame 40
Details	63	91	hsa-miR-199a-3p	CDK17	cyclin dependent kinase 17
Details	64	91	hsa-miR-199a-3p	LRRC1	leucine rich repeat containing 1
Details	65	91	hsa-miR-199a-3p	EMC1	ER membrane protein complex subunit 1
Details	66	91	hsa-miR-199a-3p	TAOK1	TAO kinase 1
Details	67	90	hsa-miR-199a-3p	APLF	aprataxin and PNKP like factor
Details	68	90	hsa-miR-199a-3p	AMZ2	archaelysin family metallopeptidase 2
Details	69	90	hsa-miR-199a-3p	DCBLD2	discoidin, CUB and LCCL domain containing 2
Details	70	90	hsa-miR-199a-3p	ITPK1	inositol-tetrakisphosphate 1-kinase
Details	71	90	hsa-miR-199a-3p	C2orf49	chromosome 2 open reading frame 49
Details	72	90	hsa-miR-199a-3p	MS4A7	membrane spanning 4-domains A7
Details	73	90	hsa-miR-199a-3p	PON2	paraoxonase 2
Details	74	90	hsa-miR-199a-3p	NLK	nemo like kinase
Details	75	89	hsa-miR-199a-3p	PROSER1	proline and serine rich 1
Details	76	89	hsa-miR-199a-3p	NEDD4	neural precursor cell expressed, developmentally down-regulated 4, E3 ubiquitin protein ligase
Details	77	89	hsa-miR-199a-3p	CSRP2	cysteine and glycine rich protein 2
Details	78	89	hsa-miR-199a-3p	PTPRZ1	protein tyrosine phosphatase, receptor type Z1
Details	79	89	hsa-miR-199a-3p	FXR1	FMR1 autosomal homolog 1

Details	80	89	hsa-miR-199a-3p	GRK3	G protein-coupled receptor kinase 3
Details	81	89	hsa-miR-199a-3p	FAM199X	family with sequence similarity 199, X-linked
Details	82	89	hsa-miR-199a-3p	CPEB4	cytoplasmic polyadenylation element binding protein 4
Details	83	89	hsa-miR-199a-3p	PPP4R2	protein phosphatase 4 regulatory subunit 2
Details	84	88	hsa-miR-199a-3p	PNRC1	proline rich nuclear receptor coactivator 1
Details	85	88	hsa-miR-199a-3p	SLC24A2	solute carrier family 24 member 2
Details	86	88	hsa-miR-199a-3p	PRPF40A	pre-mRNA processing factor 40 homolog A
Details	87	88	hsa-miR-199a-3p	SDC2	syndecan 2
Details	88	88	hsa-miR-199a-3p	DNHD1	dynein heavy chain domain 1
Details	89	88	hsa-miR-199a-3p	ESRP1	epithelial splicing regulatory protein 1
Details	90	88	hsa-miR-199a-3p	TGIF2	TGFB induced factor homeobox 2
Details	91	88	hsa-miR-199a-3p	SLITRK6	SLIT and NTRK like family member 6
Details	92	87	hsa-miR-199a-3p	RAP2A	RAP2A, member of RAS oncogene family
Details	93	87	hsa-miR-199a-3p	MCFD2	multiple coagulation factor deficiency 2
Details	94	87	hsa-miR-199a-3p	LLGL2	LLGL scribble cell polarity complex component 2
Details	95	87	hsa-miR-199a-3p	AK4	adenylate kinase 4
Details	96	87	hsa-miR-199a-3p	IFFO2	intermediate filament family orphan 2
Details	97	87	hsa-miR-199a-3p	SOS2	SOS Ras/Rho guanine nucleotide exchange factor 2
Details	98	87	hsa-miR-199a-3p	SLC44A5	solute carrier family 44 member 5
Details	99	87	hsa-miR-199a-3p	RP1	RP1, axonemal microtubule associated
Details	100	86	hsa-miR-199a-3p	SH3GLB1	SH3 domain containing GRB2 like, endophilin B1
Details	101	86	hsa-miR-199a-3p	LAMP3	lysosomal associated membrane protein 3
Details	102	86	hsa-miR-199a-3p	TMEM62	transmembrane protein 62
Details	103	86	hsa-miR-199a-3p	FUBP1	far upstream element binding protein 1
Details	104	86	hsa-miR-199a-3p	CTNNA2	catenin alpha 2
Details	105	86	hsa-miR-199a-3p	TAB2	TGF-beta activated kinase 1 (MAP3K7) binding protein 2
Details	106	86	hsa-miR-199a-3p	ANKRD61	ankyrin repeat domain 61
Details	107	86	hsa-miR-199a-3p	ATAD1	ATPase family, AAA domain containing 1
Details	108	86	hsa-miR-199a-3p	AEBP2	AE binding protein 2
Details	109	85	hsa-miR-199a-3p	ACVR2B	activin A receptor type 2B
Details	110	85	hsa-miR-199a-3p	PLEKHH1	pleckstrin homology, MyTH4 and FERM domain containing H1
Details	111	85	hsa-miR-199a-3p	VPS33A	VPS33A, CORVET/HOPS core subunit
Details	112	85	hsa-miR-199a-3p	KDM5A	lysine demethylase 5A
Details	113	85	hsa-miR-199a-3p	ADRB1	adrenoceptor beta 1
Details	114	85	hsa-miR-199a-3p	GORAB	golgin, RAB6 interacting
Details	115	85	hsa-miR-199a-3p	PTPRC	protein tyrosine phosphatase, receptor type C
Details	116	85	hsa-miR-199a-3p	NET1	neuroepithelial cell transforming 1
Details	117	84	hsa-miR-199a-3p	ADD3	adducin 3
Details	118	84	hsa-miR-199a-3p	MECP2	methyl-CpG binding protein 2
Details	119	84	hsa-miR-199a-3p	ANKRD44	ankyrin repeat domain 44
Details	120	84	hsa-miR-199a-3p	EBF1	EBF transcription factor 1
Details	121	84	hsa-miR-199a-3p	UBQLN1	ubiquilin 1
Details	122	84	hsa-miR-199a-3p	MPP7	membrane palmitoylated protein 7
Details	123	84	hsa-miR-199a-3p	CCDC85C	coiled-coil domain containing 85C
Details	124	84	hsa-miR-199a-3p	WDR47	WD repeat domain 47
Details	125	83	hsa-miR-199a-3p	SMIM8	small integral membrane protein 8

Details	126	83	hsa-miR-199a-3p	LPAR4	lysophosphatidic acid receptor 4
Details	127	83	hsa-miR-199a-3p	HIC2	HIC ZBTB transcriptional repressor 2
Details	128	83	hsa-miR-199a-3p	RBM47	RNA binding motif protein 47
Details	129	83	hsa-miR-199a-3p	NTRK2	neurotrophic receptor tyrosine kinase 2
Details	130	83	hsa-miR-199a-3p	FAM129A	family with sequence similarity 129 member A
Details	131	83	hsa-miR-199a-3p	KDM3A	lysine demethylase 3A
Details	132	83	hsa-miR-199a-3p	ZNF217	zinc finger protein 217
Details	133	83	hsa-miR-199a-3p	PIK3CB	phosphatidylinositol-4,5-bisphosphate 3-kinase catalytic subunit beta
Details	134	83	hsa-miR-199a-3p	PLAG1	PLAG1 zinc finger
Details	135	83	hsa-miR-199a-3p	CREBRF	CREB3 regulatory factor
Details	136	83	hsa-miR-199a-3p	SP1	Sp1 transcription factor
Details	137	83	hsa-miR-199a-3p	PLCB1	phospholipase C beta 1
Details	138	82	hsa-miR-199a-3p	NECTIN2	nectin cell adhesion molecule 2
Details	139	82	hsa-miR-199a-3p	MAP3K2	mitogen-activated protein kinase kinase kinase 2
Details	140	82	hsa-miR-199a-3p	TBX3	T-box 3
Details	141	82	hsa-miR-199a-3p	PSD2	pleckstrin and Sec7 domain containing 2
Details	142	82	hsa-miR-199a-3p	VAMP3	vesicle associated membrane protein 3
Details	143	81	hsa-miR-199a-3p	ITGA6	integrin subunit alpha 6
Details	144	81	hsa-miR-199a-3p	RPS6KA6	ribosomal protein S6 kinase A6
Details	145	81	hsa-miR-199a-3p	CXADR	CXADR, Ig-like cell adhesion molecule
Details	146	81	hsa-miR-199a-3p	MED12L	mediator complex subunit 12 like
Details	147	81	hsa-miR-199a-3p	ARL15	ADP ribosylation factor like GTPase 15
Details	148	81	hsa-miR-199a-3p	FN1	fibronectin 1
Details	149	81	hsa-miR-199a-3p	PDE4B	phosphodiesterase 4B
Details	150	80	hsa-miR-199a-3p	CBLB	Cbl proto-oncogene B
Details	151	80	hsa-miR-199a-3p	VLDLR	very low density lipoprotein receptor
Details	152	80	hsa-miR-199a-3p	CBLL1	Cbl proto-oncogene like 1
Details	153	80	hsa-miR-199a-3p	KLF13	Kruppel like factor 13
Details	154	80	hsa-miR-199a-3p	FGF7	fibroblast growth factor 7
Details	155	80	hsa-miR-199a-3p	CHKA	choline kinase alpha
Details	156	80	hsa-miR-199a-3p	ITGA8	integrin subunit alpha 8
Details	157	80	hsa-miR-199a-3p	CHAD	chondroadherin
Details	158	80	hsa-miR-199a-3p	UNC45A	unc-45 myosin chaperone A
Details	159	80	hsa-miR-199a-3p	MVB12B	multivesicular body subunit 12B
Details	160	80	hsa-miR-199a-3p	FBXW11	F-box and WD repeat domain containing 11
Details	161	80	hsa-miR-199a-3p	CYP1B1	cytochrome P450 family 1 subfamily B member 1
Details	162	80	hsa-miR-199a-3p	CHSY3	chondroitin sulfate synthase 3
Details	163	79	hsa-miR-199a-3p	PDE5A	phosphodiesterase 5A
Details	164	79	hsa-miR-199a-3p	SLC7A11	solute carrier family 7 member 11
Details	165	79	hsa-miR-199a-3p	UPRT	uracil phosphoribosyltransferase homolog
Details	166	79	hsa-miR-199a-3p	RNGTT	RNA guanylyltransferase and 5'-phosphatase
Details	167	79	hsa-miR-199a-3p	C9orf170	chromosome 9 open reading frame 170
Details	168	79	hsa-miR-199a-3p	CETN3	centrin 3
Details	169	79	hsa-miR-199a-3p	RABEP1	rabaptin, RAB GTPase binding effector protein 1
Details	170	79	hsa-miR-199a-3p	MTOR	mechanistic target of rapamycin kinase
Details	171	79	hsa-miR-199a-3p	SEC16B	SEC16 homolog B, endoplasmic reticulum export factor
Details	172	79	hsa-miR-199a-3p	EPB41L5	erythrocyte membrane protein band 4.1 like 5

Details	173	79	hsa-miR-199a-3p	ACOX1	acyl-CoA oxidase 1
Details	174	78	hsa-miR-199a-3p	SLC49A4	solute carrier family 49 member 4
Details	175	78	hsa-miR-199a-3p	HS3ST5	heparan sulfate-glucosamine 3-sulfotransferase 5
Details	176	78	hsa-miR-199a-3p	PDE8A	phosphodiesterase 8A
Details	177	78	hsa-miR-199a-3p	CD151	CD151 molecule (Raph blood group)
Details	178	78	hsa-miR-199a-3p	LCOR	ligand dependent nuclear receptor corepressor
Details	179	78	hsa-miR-199a-3p	KCTD16	potassium channel tetramerization domain containing 16
Details	180	78	hsa-miR-199a-3p	CD44	CD44 molecule (Indian blood group)
Details	181	78	hsa-miR-199a-3p	GNPTAB	N-acetylglucosamine-1-phosphate transferase subunits alpha and beta
Details	182	77	hsa-miR-199a-3p	AREL1	apoptosis resistant E3 ubiquitin protein ligase 1
Details	183	77	hsa-miR-199a-3p	LRRC17	leucine rich repeat containing 17
Details	184	77	hsa-miR-199a-3p	ZBTB43	zinc finger and BTB domain containing 43
Details	185	77	hsa-miR-199a-3p	CA5B	carbonic anhydrase 5B
Details	186	77	hsa-miR-199a-3p	UXS1	UDP-glucuronate decarboxylase 1
Details	187	77	hsa-miR-199a-3p	COLCA1	colorectal cancer associated 1
Details	188	77	hsa-miR-199a-3p	SBNO1	strawberry notch homolog 1
Details	189	77	hsa-miR-199a-3p	RABGAP1	RAB GTPase activating protein 1
Details	190	77	hsa-miR-199a-3p	TMEM220	transmembrane protein 220
Details	191	76	hsa-miR-199a-3p	KIDINS220	kinase D interacting substrate 220
Details	192	76	hsa-miR-199a-3p	ELAVL2	ELAV like RNA binding protein 2
Details	193	76	hsa-miR-199a-3p	YAP1	Yes associated protein 1
Details	194	76	hsa-miR-199a-3p	CABLES1	Cdk5 and Abl enzyme substrate 1
Details	195	76	hsa-miR-199a-3p	ANO5	anoctamin 5
Details	196	75	hsa-miR-199a-3p	DDIT4	DNA damage inducible transcript 4
Details	197	75	hsa-miR-199a-3p	STARD9	StAR related lipid transfer domain containing 9
Details	198	75	hsa-miR-199a-3p	GOLIM4	golgi integral membrane protein 4
Details	199	75	hsa-miR-199a-3p	IMMT	inner membrane mitochondrial protein
Details	200	75	hsa-miR-199a-3p	LOC100144595	uncharacterized LOC100144595
Details	201	75	hsa-miR-199a-3p	PHYHIPL	phytanoyl-CoA 2-hydroxylase interacting protein like
Details	202	75	hsa-miR-199a-3p	YES1	YES proto-oncogene 1, Src family tyrosine kinase
Details	203	75	hsa-miR-199a-3p	CFL2	cofilin 2
Details	204	75	hsa-miR-199a-3p	NUFIP2	nuclear FMR1 interacting protein 2
Details	205	75	hsa-miR-199a-3p	CDK5R1	cyclin dependent kinase 5 regulatory subunit 1
Details	206	75	hsa-miR-199a-3p	COL4A5	collagen type IV alpha 5 chain
Details	207	75	hsa-miR-199a-3p	CLDN8	claudin 8
Details	208	75	hsa-miR-199a-3p	ST6GAL2	ST6 beta-galactoside alpha-2,6-sialyltransferase 2
Details	209	75	hsa-miR-199a-3p	ZBTB20	zinc finger and BTB domain containing 20
Details	210	74	hsa-miR-199a-3p	CAPRIN1	cell cycle associated protein 1
Details	211	74	hsa-miR-199a-3p	CNOT7	CCR4-NOT transcription complex subunit 7
Details	212	74	hsa-miR-199a-3p	HVCN1	hydrogen voltage gated channel 1
Details	213	74	hsa-miR-199a-3p	TMSB4X	thymosin beta 4 X-linked
Details	214	74	hsa-miR-199a-3p	EIF3M	eukaryotic translation initiation factor 3 subunit M
Details	215	74	hsa-miR-199a-3p	HNMT	histamine N-methyltransferase

Details	216	73	hsa-miR-199a-3p	PAQR3	progestin and adipoQ receptor family member 3
Details	217	73	hsa-miR-199a-3p	NETO1	neuropilin and tolloid like 1
Details	218	73	hsa-miR-199a-3p	HACE1	HECT domain and ankyrin repeat containing E3 ubiquitin protein ligase 1
Details	219	73	hsa-miR-199a-3p	ROCK2	Rho associated coiled-coil containing protein kinase 2
Details	220	73	hsa-miR-199a-3p	DPAGT1	dolichyl-phosphate N-acetylglucosaminephosphotransferase 1
Details	221	73	hsa-miR-199a-3p	FMN1	formin 1
Details	222	73	hsa-miR-199a-3p	NXPH1	neurexophilin 1
Details	223	72	hsa-miR-199a-3p	MKRN1	makorin ring finger protein 1
Details	224	72	hsa-miR-199a-3p	PYHIN1	pyrin and HIN domain family member 1
Details	225	72	hsa-miR-199a-3p	MEDAG	mesenteric estrogen dependent adipogenesis
Details	226	72	hsa-miR-199a-3p	ATP6V1C2	ATPase H+ transporting V1 subunit C2
Details	227	72	hsa-miR-199a-3p	TAF2	TATA-box binding protein associated factor 2
Details	228	72	hsa-miR-199a-3p	GGNBP2	gametogenetin binding protein 2
Details	229	72	hsa-miR-199a-3p	KHDC4	KH domain containing 4, pre-mRNA splicing factor
Details	230	72	hsa-miR-199a-3p	PPP2R2A	protein phosphatase 2 regulatory subunit Balpha
Details	231	72	hsa-miR-199a-3p	ZBTB18	zinc finger and BTB domain containing 18
Details	232	72	hsa-miR-199a-3p	PTPRU	protein tyrosine phosphatase, receptor type U
Details	233	71	hsa-miR-199a-3p	DOLPP1	dolichyldiphosphatase 1
Details	234	71	hsa-miR-199a-3p	SYNPO2	synaptopodin 2
Details	235	71	hsa-miR-199a-3p	SEMA3E	semaphorin 3E
Details	236	71	hsa-miR-199a-3p	PLGLB2	plasminogen-like B2
Details	237	71	hsa-miR-199a-3p	MAGT1	magnesium transporter 1
Details	238	71	hsa-miR-199a-3p	CNIH2	cornichon family AMPA receptor auxiliary protein 2
Details	239	71	hsa-miR-199a-3p	TACC2	transforming acidic coiled-coil containing protein 2
Details	240	71	hsa-miR-199a-3p	NRBP2	nuclear receptor binding protein 2
Details	241	71	hsa-miR-199a-3p	RALGPS2	Ral GEF with PH domain and SH3 binding motif 2
Details	242	71	hsa-miR-199a-3p	WNK1	WNK lysine deficient protein kinase 1
Details	243	71	hsa-miR-199a-3p	RSBN1	round spermatid basic protein 1
Details	244	70	hsa-miR-199a-3p	MDGA2	MAM domain containing glycosylphosphatidylinositol anchor 2
Details	245	70	hsa-miR-199a-3p	KCTD7	potassium channel tetramerization domain containing 7
Details	246	70	hsa-miR-199a-3p	DIMT1	DIM1 dimethyladenosine transferase 1 homolog
Details	247	70	hsa-miR-199a-3p	TRIP11	thyroid hormone receptor interactor 11
Details	248	70	hsa-miR-199a-3p	EOGT	EGF domain specific O-linked N-acetylglucosamine transferase
Details	249	70	hsa-miR-199a-3p	HYPK	huntingtin interacting protein K
Details	250	70	hsa-miR-199a-3p	PTPRE	protein tyrosine phosphatase, receptor type E
Details	251	70	hsa-miR-199a-3p	PHKA1	phosphorylase kinase regulatory subunit alpha 1
Details	252	70	hsa-miR-199a-3p	WHAMM	WAS protein homolog associated with actin, golgi membranes and microtubules
Details	253	69	hsa-miR-199a-3p	WDR7	WD repeat domain 7

Details	254	69	hsa-miR-199a-3p	MAPRE1	microtubule associated protein RP/EB family member 1
Details	255	69	hsa-miR-199a-3p	FGL2	fibrinogen like 2
Details	256	69	hsa-miR-199a-3p	GIP	gastric inhibitory polypeptide
Details	257	69	hsa-miR-199a-3p	ZEB1	zinc finger E-box binding homeobox 1
Details	258	69	hsa-miR-199a-3p	DLX2	distal-less homeobox 2
Details	259	69	hsa-miR-199a-3p	ZNF740	zinc finger protein 740
Details	260	69	hsa-miR-199a-3p	ERO1A	endoplasmic reticulum oxidoreductase 1 alpha
Details	261	69	hsa-miR-199a-3p	GCNT2	glucosaminyl (N-acetyl) transferase 2 (I blood group)
Details	262	68	hsa-miR-199a-3p	TCAF2	TRPM8 channel associated factor 2
Details	263	68	hsa-miR-199a-3p	PLGLB1	plasminogen-like B1
Details	264	68	hsa-miR-199a-3p	FCGR3A	Fc fragment of IgG receptor IIIa
Details	265	68	hsa-miR-199a-3p	NACC2	NACC family member 2
Details	266	68	hsa-miR-199a-3p	ZCCHC17	zinc finger CCHC-type containing 17
Details	267	67	hsa-miR-199a-3p	DLX5	distal-less homeobox 5
Details	268	67	hsa-miR-199a-3p	TENM1	teneurin transmembrane protein 1
Details	269	67	hsa-miR-199a-3p	SESN3	sestrin 3
Details	270	67	hsa-miR-199a-3p	ARHGEF12	Rho guanine nucleotide exchange factor 12
Details	271	67	hsa-miR-199a-3p	GREM1	gremlin 1, DAN family BMP antagonist
Details	272	67	hsa-miR-199a-3p	KPNB1	karyopherin subunit beta 1
Details	273	67	hsa-miR-199a-3p	RAB3D	RAB3D, member RAS oncogene family
Details	274	66	hsa-miR-199a-3p	GPBP1L1	GC-rich promoter binding protein 1 like 1
Details	275	66	hsa-miR-199a-3p	PDGFRA	platelet derived growth factor receptor alpha
Details	276	66	hsa-miR-199a-3p	EML4	EMAP like 4
Details	277	66	hsa-miR-199a-3p	FCGR3B	Fc fragment of IgG receptor IIIb
Details	278	66	hsa-miR-199a-3p	DELE1	DAP3 binding cell death enhancer 1
Details	279	66	hsa-miR-199a-3p	FAM110C	family with sequence similarity 110 member C
Details	280	66	hsa-miR-199a-3p	PCMTD1	protein-L-isoaspartate (D-aspartate) O-methyltransferase domain containing 1
Details	281	66	hsa-miR-199a-3p	ZBTB25	zinc finger and BTB domain containing 25
Details	282	66	hsa-miR-199a-3p	SORL1	sortilin related receptor 1
Details	283	66	hsa-miR-199a-3p	ARHGAP21	Rho GTPase activating protein 21
Details	284	65	hsa-miR-199a-3p	CCL28	C-C motif chemokine ligand 28
Details	285	65	hsa-miR-199a-3p	UPF2	UPF2, regulator of nonsense mediated mRNA decay
Details	286	65	hsa-miR-199a-3p	FOXQ1	forkhead box Q1
Details	287	65	hsa-miR-199a-3p	EXOC6B	exocyst complex component 6B
Details	288	65	hsa-miR-199a-3p	PIP5K1B	phosphatidylinositol-4-phosphate 5-kinase type 1 beta
Details	289	65	hsa-miR-199a-3p	LOX	lysyl oxidase
Details	290	65	hsa-miR-199a-3p	DCP2	decapping mRNA 2
Details	291	65	hsa-miR-199a-3p	TEAD1	TEA domain transcription factor 1
Details	292	65	hsa-miR-199a-3p	PCDHB12	protocadherin beta 12
Details	293	65	hsa-miR-199a-3p	MCCC2	methylcrotonoyl-CoA carboxylase 2
Details	294	64	hsa-miR-199a-3p	TCTA	T cell leukemia translocation altered
Details	295	64	hsa-miR-199a-3p	LPAR5	lysophosphatidic acid receptor 5
Details	296	64	hsa-miR-199a-3p	MFSD6	major facilitator superfamily domain containing 6
Details	297	64	hsa-miR-199a-3p	NCOA1	nuclear receptor coactivator 1
Details	298	64	hsa-miR-199a-3p	PNOC	prepronociceptin
Details	299	63	hsa-miR-199a-3p	PTER	phosphotriesterase related

Details	300	63	hsa-miR-199a-3p	ZNF614	zinc finger protein 614
Details	301	63	hsa-miR-199a-3p	DIO2	iodothyronine deiodinase 2
Details	302	63	hsa-miR-199a-3p	UBE2W	ubiquitin conjugating enzyme E2 W
Details	303	63	hsa-miR-199a-3p	ATP6V1A	ATPase H+ transporting V1 subunit A
Details	304	63	hsa-miR-199a-3p	FLRT3	fibronectin leucine rich transmembrane protein 3
Details	305	63	hsa-miR-199a-3p	PLEKHA3	pleckstrin homology domain containing A3
Details	306	63	hsa-miR-199a-3p	BRWD3	bromodomain and WD repeat domain containing 3
Details	307	63	hsa-miR-199a-3p	ZBTB4	zinc finger and BTB domain containing 4
Details	308	63	hsa-miR-199a-3p	CCDC28B	coiled-coil domain containing 28B
Details	309	63	hsa-miR-199a-3p	TMED5	transmembrane p24 trafficking protein 5
Details	310	62	hsa-miR-199a-3p	SMOC1	SPARC related modular calcium binding 1
Details	311	62	hsa-miR-199a-3p	KTN1	kinectin 1
Details	312	62	hsa-miR-199a-3p	HGF	hepatocyte growth factor
Details	313	62	hsa-miR-199a-3p	XKR9	XK related 9
Details	314	62	hsa-miR-199a-3p	CLCC1	chloride channel CLIC like 1
Details	315	62	hsa-miR-199a-3p	CHMP5	charged multivesicular body protein 5
Details	316	62	hsa-miR-199a-3p	ARG2	arginase 2
Details	317	62	hsa-miR-199a-3p	PEX13	peroxisomal biogenesis factor 13
Details	318	62	hsa-miR-199a-3p	ARL6IP1	ADP ribosylation factor like GTPase 6 interacting protein 1
Details	319	62	hsa-miR-199a-3p	SECISBP2L	SECIS binding protein 2 like
Details	320	62	hsa-miR-199a-3p	ANKRD17	ankyrin repeat domain 17
Details	321	62	hsa-miR-199a-3p	TNIK	TRAF2 and NCK interacting kinase
Details	322	62	hsa-miR-199a-3p	TMEM87A	transmembrane protein 87A
Details	323	61	hsa-miR-199a-3p	KMT2A	lysine methyltransferase 2A
Details	324	61	hsa-miR-199a-3p	CALD1	caldesmon 1
Details	325	61	hsa-miR-199a-3p	FAM20B	FAM20B, glycosaminoglycan xylosylkinase
Details	326	61	hsa-miR-199a-3p	SPIRE1	spire type actin nucleation factor 1
Details	327	61	hsa-miR-199a-3p	KLF12	Kruppel like factor 12
Details	328	61	hsa-miR-199a-3p	CCSER1	coiled-coil serine rich protein 1
Details	329	61	hsa-miR-199a-3p	FDX1	ferredoxin 1
Details	330	61	hsa-miR-199a-3p	TSPAN3	tetraspanin 3
Details	331	61	hsa-miR-199a-3p	PALD1	phosphatase domain containing paladin 1
Details	332	61	hsa-miR-199a-3p	IRAK3	interleukin 1 receptor associated kinase 3
Details	333	61	hsa-miR-199a-3p	PHF6	PHD finger protein 6
Details	334	61	hsa-miR-199a-3p	FOXP1	forkhead box P1
Details	335	61	hsa-miR-199a-3p	ACVR1C	activin A receptor type 1C
Details	336	61	hsa-miR-199a-3p	BBX	BBX, HMG-box containing
Details	337	61	hsa-miR-199a-3p	TRIM5	tripartite motif containing 5
Details	338	60	hsa-miR-199a-3p	SLC33A1	solute carrier family 33 member 1
Details	339	60	hsa-miR-199a-3p	ID4	inhibitor of DNA binding 4, HLH protein
Details	340	60	hsa-miR-199a-3p	PRKCB	protein kinase C beta
Details	341	60	hsa-miR-199a-3p	MAP3K1	mitogen-activated protein kinase kinase kinase 1
Details	342	60	hsa-miR-199a-3p	INO80D	INO80 complex subunit D
Details	343	60	hsa-miR-199a-3p	TNFRSF19	TNF receptor superfamily member 19
Details	344	60	hsa-miR-199a-3p	MYSM1	Myb like, SWIRM and MPN domains 1
Details	345	60	hsa-miR-199a-3p	PDE7B	phosphodiesterase 7B
Details	346	60	hsa-miR-199a-3p	ARHGAP20	Rho GTPase activating protein 20
Details	347	60	hsa-miR-199a-3p	UBE2J1	ubiquitin conjugating enzyme E2 J1

Details	348	60	hsa-miR-199a-3p	NUTF2	nuclear transport factor 2
Details	349	60	hsa-miR-199a-3p	LONP2	lon peptidase 2, peroxisomal
Details	350	60	hsa-miR-199a-3p	SLC16A12	solute carrier family 16 member 12
Details	351	60	hsa-miR-199a-3p	IPPK	inositol-pentakisphosphate 2-kinase
Details	352	59	hsa-miR-199a-3p	TWNK	twinkle mtDNA helicase
Details	353	59	hsa-miR-199a-3p	RNFT1	ring finger protein, transmembrane 1
Details	354	59	hsa-miR-199a-3p	NAP1L1	nucleosome assembly protein 1 like 1
Details	355	59	hsa-miR-199a-3p	CELF2	CUGBP Elav-like family member 2
Details	356	59	hsa-miR-199a-3p	ABHD4	abhydrolase domain containing 4
Details	357	59	hsa-miR-199a-3p	WAPL	WAPL cohesin release factor
Details	358	58	hsa-miR-199a-3p	BCL2L13	BCL2 like 13
Details	359	58	hsa-miR-199a-3p	DONSON	downstream neighbor of SON
Details	360	58	hsa-miR-199a-3p	GIMAP2	GTPase, IMAP family member 2
Details	361	58	hsa-miR-199a-3p	ENOX2	ecto-NOX disulfide-thiol exchanger 2
Details	362	58	hsa-miR-199a-3p	HAT1	histone acetyltransferase 1
Details	363	58	hsa-miR-199a-3p	ATL1	atlastin GTPase 1
Details	364	58	hsa-miR-199a-3p	SLC5A7	solute carrier family 5 member 7
Details	365	58	hsa-miR-199a-3p	LIG3	DNA ligase 3
Details	366	58	hsa-miR-199a-3p	RUNDC1	RUN domain containing 1
Details	367	58	hsa-miR-199a-3p	CXCL11	C-X-C motif chemokine ligand 11
Details	368	58	hsa-miR-199a-3p	TBC1D12	TBC1 domain family member 12
Details	369	58	hsa-miR-199a-3p	SNX18	sorting nexin 18
Details	370	58	hsa-miR-199a-3p	CAMK4	calcium/calmodulin dependent protein kinase IV
Details	371	58	hsa-miR-199a-3p	PRKCE	protein kinase C epsilon
Details	372	58	hsa-miR-199a-3p	CR1	complement C3b/C4b receptor 1 (Knops blood group)
Details	373	58	hsa-miR-199a-3p	SLC38A1	solute carrier family 38 member 1
Details	374	58	hsa-miR-199a-3p	MYO10	myosin X
Details	375	57	hsa-miR-199a-3p	DCLK1	doublecortin like kinase 1
Details	376	57	hsa-miR-199a-3p	SEC62	SEC62 homolog, preprotein translocation factor
Details	377	57	hsa-miR-199a-3p	ZNF736	zinc finger protein 736
Details	378	57	hsa-miR-199a-3p	ST8SIA4	ST8 alpha-N-acetyl-neuraminide alpha-2,8-sialyltransferase 4
Details	379	57	hsa-miR-199a-3p	SLC7A1	solute carrier family 7 member 1
Details	380	57	hsa-miR-199a-3p	CISD2	CDGSH iron sulfur domain 2
Details	381	57	hsa-miR-199a-3p	SYT16	synaptotagmin 16
Details	382	57	hsa-miR-199a-3p	P2RY12	purinergic receptor P2Y12
Details	383	57	hsa-miR-199a-3p	C6orf62	chromosome 6 open reading frame 62
Details	384	57	hsa-miR-199a-3p	KPNA6	karyopherin subunit alpha 6
Details	385	56	hsa-miR-199a-3p	MTF2	metal response element binding transcription factor 2
Details	386	56	hsa-miR-199a-3p	SLC22A15	solute carrier family 22 member 15
Details	387	56	hsa-miR-199a-3p	BRCA1	BRCA1, DNA repair associated
Details	388	56	hsa-miR-199a-3p	SNN	stannin
Details	389	56	hsa-miR-199a-3p	LZTS3	leucine zipper tumor suppressor family member 3
Details	390	56	hsa-miR-199a-3p	ZNF227	zinc finger protein 227
Details	391	56	hsa-miR-199a-3p	NECTIN3	nectin cell adhesion molecule 3
Details	392	56	hsa-miR-199a-3p	ITPKC	inositol-trisphosphate 3-kinase C
Details	393	56	hsa-miR-199a-3p	KCMF1	potassium channel modulatory factor 1

Details	394	56	hsa-miR-199a-3p	KCNH2	potassium voltage-gated channel subfamily H member 2
Details	395	56	hsa-miR-199a-3p	B3GALNT2	beta-1,3-N-acetylgalactosaminyltransferase 2
Details	396	56	hsa-miR-199a-3p	TANC1	tetratricopeptide repeat, ankyrin repeat and coiled-coil containing 1
Details	397	56	hsa-miR-199a-3p	SUMO3	small ubiquitin-like modifier 3
Details	398	56	hsa-miR-199a-3p	NCOA4	nuclear receptor coactivator 4
Details	399	55	hsa-miR-199a-3p	N4BP2L1	NEDD4 binding protein 2 like 1
Details	400	55	hsa-miR-199a-3p	RGS4	regulator of G protein signaling 4
Details	401	55	hsa-miR-199a-3p	GFOD2	glucose-fructose oxidoreductase domain containing 2
Details	402	55	hsa-miR-199a-3p	RIMBP2	RIMS binding protein 2
Details	403	55	hsa-miR-199a-3p	CABYR	calcium binding tyrosine phosphorylation regulated
Details	404	55	hsa-miR-199a-3p	DUSP5	dual specificity phosphatase 5
Details	405	55	hsa-miR-199a-3p	NABP1	nucleic acid binding protein 1
Details	406	55	hsa-miR-199a-3p	FAM133A	family with sequence similarity 133 member A
Details	407	55	hsa-miR-199a-3p	MPRIP	myosin phosphatase Rho interacting protein
Details	408	55	hsa-miR-199a-3p	WDR41	WD repeat domain 41
Details	409	55	hsa-miR-199a-3p	THAP9	THAP domain containing 9
Details	410	55	hsa-miR-199a-3p	CNEP1R1	CTD nuclear envelope phosphatase 1 regulatory subunit 1
Details	411	55	hsa-miR-199a-3p	CCDC141	coiled-coil domain containing 141
Details	412	55	hsa-miR-199a-3p	THAP2	THAP domain containing 2
Details	413	55	hsa-miR-199a-3p	ARL6IP6	ADP ribosylation factor like GTPase 6 interacting protein 6
Details	414	55	hsa-miR-199a-3p	ZNF749	zinc finger protein 749
Details	415	55	hsa-miR-199a-3p	SLC22A5	solute carrier family 22 member 5
Details	416	54	hsa-miR-199a-3p	CNR1	cannabinoid receptor 1
Details	417	54	hsa-miR-199a-3p	TMEM218	transmembrane protein 218
Details	418	54	hsa-miR-199a-3p	ABL2	ABL proto-oncogene 2, non-receptor tyrosine kinase
Details	419	54	hsa-miR-199a-3p	PXN	paxillin
Details	420	54	hsa-miR-199a-3p	BEND7	BEN domain containing 7
Details	421	54	hsa-miR-199a-3p	HBS1L	HBS1 like translational GTPase
Details	422	54	hsa-miR-199a-3p	KDR	kinase insert domain receptor
Details	423	54	hsa-miR-199a-3p	MEIS2	Meis homeobox 2
Details	424	54	hsa-miR-199a-3p	TENT5D	terminal nucleotidyltransferase 5D
Details	425	54	hsa-miR-199a-3p	KSR2	kinase suppressor of ras 2
Details	426	54	hsa-miR-199a-3p	DTNA	dystrobrevin alpha
Details	427	54	hsa-miR-199a-3p	DNAJC30	DnaJ heat shock protein family (Hsp40) member C30
Details	428	54	hsa-miR-199a-3p	SRA1	steroid receptor RNA activator 1
Details	429	53	hsa-miR-199a-3p	SEPT14	septin 14
Details	430	53	hsa-miR-199a-3p	TRIM71	tripartite motif containing 71
Details	431	53	hsa-miR-199a-3p	GABRP	gamma-aminobutyric acid type A receptor pi subunit
Details	432	53	hsa-miR-199a-3p	DNAJC18	DnaJ heat shock protein family (Hsp40) member C18
Details	433	53	hsa-miR-199a-3p	MBL2	mannose binding lectin 2
Details	434	53	hsa-miR-199a-3p	PLCXD3	phosphatidylinositol specific phospholipase C X domain containing 3
Details	435	53	hsa-miR-199a-3p	UBR2	ubiquitin protein ligase E3 component n-

					recognin 2
Details	436	53	hsa-miR-199a-3p	MLLT6	MLLT6, PHD finger containing
Details	437	53	hsa-miR-199a-3p	OPN5	opsin 5
Details	438	53	hsa-miR-199a-3p	SRD5A3	steroid 5 alpha-reductase 3
Details	439	53	hsa-miR-199a-3p	SYNJ1	synaptojanin 1
Details	440	53	hsa-miR-199a-3p	PIGB	phosphatidylinositol glycan anchor biosynthesis class B
Details	441	52	hsa-miR-199a-3p	EPAS1	endothelial PAS domain protein 1
Details	442	52	hsa-miR-199a-3p	TRMT61B	tRNA methyltransferase 61B
Details	443	52	hsa-miR-199a-3p	FAM76B	family with sequence similarity 76 member B
Details	444	52	hsa-miR-199a-3p	OSTM1	osteoclastogenesis associated transmembrane protein 1
Details	445	52	hsa-miR-199a-3p	RAB6C	RAB6C, member RAS oncogene family
Details	446	52	hsa-miR-199a-3p	CLEC12B	C-type lectin domain family 12 member B
Details	447	52	hsa-miR-199a-3p	SCUBE3	signal peptide, CUB domain and EGF like domain containing 3
Details	448	52	hsa-miR-199a-3p	FUT9	fucosyltransferase 9
Details	449	52	hsa-miR-199a-3p	IL1RL1	interleukin 1 receptor like 1
Details	450	52	hsa-miR-199a-3p	HSDL1	hydroxysteroid dehydrogenase like 1
Details	451	52	hsa-miR-199a-3p	DPF3	double PHD fingers 3
Details	452	52	hsa-miR-199a-3p	CDC42BPB	CDC42 binding protein kinase beta
Details	453	52	hsa-miR-199a-3p	GPAT3	glycerol-3-phosphate acyltransferase 3
Details	454	52	hsa-miR-199a-3p	ID2	inhibitor of DNA binding 2
Details	455	52	hsa-miR-199a-3p	MAN1A2	mannosidase alpha class 1A member 2
Details	456	51	hsa-miR-199a-3p	SIK2	salt inducible kinase 2
Details	457	51	hsa-miR-199a-3p	CCDC88C	coiled-coil domain containing 88C
Details	458	51	hsa-miR-199a-3p	GFM1	G elongation factor mitochondrial 1
Details	459	51	hsa-miR-199a-3p	NDST3	N-deacetylase and N-sulfotransferase 3
Details	460	51	hsa-miR-199a-3p	CMIP	c-Maf inducing protein
Details	461	51	hsa-miR-199a-3p	UQCRB	ubiquinol-cytochrome c reductase binding protein
Details	462	51	hsa-miR-199a-3p	RAB6D	RAB6D, member RAS oncogene family
Details	463	51	hsa-miR-199a-3p	KLLN	killin, p53 regulated DNA replication inhibitor
Details	464	51	hsa-miR-199a-3p	DIRAS2	DIRAS family GTPase 2
Details	465	51	hsa-miR-199a-3p	IRF2BP2	interferon regulatory factor 2 binding protein 2
Details	466	51	hsa-miR-199a-3p	RNF19A	ring finger protein 19A, RBR E3 ubiquitin protein ligase
Details	467	50	hsa-miR-199a-3p	EDEM3	ER degradation enhancing alpha-mannosidase like protein 3
Details	468	50	hsa-miR-199a-3p	MARC1	mitochondrial amidoxime reducing component 1
Details	469	50	hsa-miR-199a-3p	IL13RA1	interleukin 13 receptor subunit alpha 1
Details	470	50	hsa-miR-199a-3p	STC2	stanniocalcin 2
Details	471	50	hsa-miR-199a-3p	RAP2B	RAP2B, member of RAS oncogene family
Details	472	50	hsa-miR-199a-3p	MARS2	methionyl-tRNA synthetase 2, mitochondrial
Details	473	50	hsa-miR-199a-3p	LRRTM2	leucine rich repeat transmembrane neuronal 2
Details	474	50	hsa-miR-199a-3p	SYPL1	synaptophysin like 1
Details	475	50	hsa-miR-199a-3p	RAB6A	RAB6A, member RAS oncogene family
Details	476	50	hsa-miR-199a-3p	LUC7L3	LUC7 like 3 pre-mRNA splicing factor
Details	477	50	hsa-miR-199a-3p	IMP3	IMP3, U3 small nucleolar ribonucleoprotein

Supplementary Table-6. The aberrantly expressed genes in ALI lung tissues

Gene	Gene Description	Change fold	P Value	Expression
TNFA	Tumor necrosis factor alpha	13.5	0.00025	Upregulation
S100A8	S100 calcium binding protein A8	13.1	0.00038	Upregulation
IL6	Interleukin-6	12.7	0.0022	Upregulation
CtBP2	C-terminal binding protein 2	12.3	0.0075	Upregulation
TIPARP	TCDD inducible poly (ADP-Ribose) polymerase	12.0	0.0062	Upregulation
TNFSF9	TNF superfamily member 9	11.6	0.0024	Upregulation
TGB1	Transforming growth factor beta 1	11.3	0.0031	Upregulation
IL1B	Interleukin1-beta	10.9	0.0044	Upregulation
NLRP1	NLR family pyrin domain containing 1	10.6	0.0013	Upregulation
IL18	Interleukin-18	10.4	0.0022	Upregulation
S100A9	S100 calcium binding protein A9	10.1	0.00055	Upregulation
ICAM1	Intercellular adhesion molecule 1	9.9	0.0052	Upregulation
NLRP3	NLR family pyrin domain containing 3	9.6	0.00035	Upregulation
MEAF6	MYST/ESA1 associated factor 6	9.3	0.00021	Upregulation
SYCP1	Synaptonemal complex protein 1	9.1	0.0011	Upregulation
ZNF436	Zinc finger protein 436	8.9	0.0044	Upregulation
CtBP1	C-terminal binding protein 1	8.8	0.0032	Upregulation
ZNF687	Zing finger protein 687	8.8	0.00017	Upregulation
NABP1	Nucleic acid binding protein 1	8.5	0.0043	Upregulation
INPP1	Inositol polyphosphate 1-phosphatase	8.4	0.00021	Upregulation
NOL6	Nucleolar protein 6	8.4	0.0022	Upregulation
KYAT1	Kynurenine aminotransferase 1	8.2	0.0084	Upregulation
IL15	Interleukin-15	8.1	0.0045	Upregulation
BAFF	B-cell activating factor	8.0	0.0028	Upregulation
IFNG	Interferon gamma	8.0	0.0054	Upregulation
SOD1	Superoxide dismutase 1	7.9	0.0072	Upregulation

SOD2	Superoxide dismutase 1	7.8	0.0045	Upregulation
NOD1	Nucleotide binding oligomerization domain containing 1	7.8	0.0087	Upregulation
CCR6	C-C Motif chemokine receptor 6	7.8	0.0047	Upregulation
SOCS3	Suppressor of cytokine signaling 3	7.7	0.0082	Upregulation
IRF5	Interferon regulatory factor 5	7.6	0.0091	Upregulation
ATN1	Atrophin 1	7.5	0.0031	Upregulation
RIPK1	Receptor interacting serine/threonine kinase 1	7.5	0.0023	Upregulation
ADAM17	DAM metallopeptidase domain 17	7.5	0.0028	Upregulation
DDX58	DExD/H-Box helicase 58	7.4	0.0045	Upregulation
SLC11A1	Solute carrier family 11 member 1	7.3	0.0026	Upregulation
NLRC4	NLR family CARD domain containing 4	7.3	0.0035	Upregulation
NPL	N-Acetylneuraminate pyruvate lyase	7.3	0.0091	Upregulation
MYD88	MYD88 innate immune signal transduction adaptor	7.2	0.0021	Upregulation
HSP90AB1	Heat shock protein 90 Alpha family class B member 1	7.2	0.0034	Upregulation
RNF31	Ring finger protein 31	7.2	0.0073	Upregulation
DEFA5	Defensin alpha 5	7.2	0.0072	Upregulation
DEFA6	Defensin alpha 6	7.2	0.0046	Upregulation
HSPA2	Heat shock protein family A (Hsp70) member 2	7.1	0.0038	Upregulation
HOGA1	4-Hydroxy-2-Oxoglutarate Aldolase 1	7.1	0.0036	Upregulation
STAT3	Signal transducer and activator of transcription 3	7.1	0.0047	Upregulation
RPL6	Ribosomal Protein L6	7.1	0.00018	Upregulation
PPARG	Peroxisome proliferator activated receptor gamma	7.0	0.0079	Upregulation
SLC6A4	Solute carrier family 6 member 4	7.0	0.0093	Upregulation

COCH	Cochlin	7.0	0.0023	Upregulation
ITLN1	Intelectin 1	7.0	0.0048	Upregulation
BTNL2	Butyrophilin Like 2	6.9	0.0065	Upregulation
AIF1	Allograft inflammatory factor 1	6.9	0.0037	Upregulation
LTB	Lymphotoxin Beta	6.9	0.0038	Upregulation
CASP1	Caspase 1	6.9	0.0041	Upregulation
NOS3	Nitric Oxide Synthase 3	6.9	0.0055	Upregulation
TGIF1	TGFB induced factor homeobox 1	6.8	0.0067	Upregulation
IFNGR2	Interferon gamma receptor 2	6.7	0.0024	Upregulation
S100A6	S100 Calcium Binding Protein A6	6.7	0.0078	Upregulation
S100A7	S100 Calcium Binding Protein A7	6.6	0.0056	Upregulation
NQO1	NAD(P)H quinone dehydrogenase 1	6.5	0.0033	Upregulation
GSTT1	Glutathione S-transferase theta 1	6.5	0.0078	Upregulation
UCP2	Uncoupling protein 2	6.4	0.0052	Upregulation
BRD4	Bromodomain Containing 4	6.4	0.0032	Upregulation
BMP6	Bone morphogenetic protein 6	6.3	0.0053	Upregulation
BMP1	Bone morphogenetic protein 1	6.2	0.0036	Upregulation
CASP8	Caspase 8	6.0	0.0048	Upregulation
XDH	Xanthine dehydrogenase	5.8	0.0083	Upregulation
THSD4	Thrombospondin type 1 domain containing 4	5.6	0.0034	Upregulation
KLF11	Kruppel like factor 11	5.6	0.0065	Upregulation
BMP2	Bone morphogenetic protein 2	5.5	0.0024	Upregulation
GDF5	Growth differentiation factor	5.5	0.0093	Upregulation
ACVRL1	Activin A receptor like type 1	5.5	0.0084	Upregulation
GDNF	Glial cell derived neurotrophic factor	5.4	0.0035	Upregulation
NFKB1	Nuclear factor Kappa B subunit 1	5.2	0.0013	Upregulation
RELA	RELA proto-oncogene, NF-KB subunit	5.2	0.0034	Upregulation
CARD16	Caspase recruitment domain family member 16	4.9	0.0062	Upregulation
AKT2	AKT Serine/Threonine kinase 2	4.8	0.0074	Upregulation

TBP	TATA-Box binding protein	4.6	0.0025	Upregulation
CUL1	Cullin 1	4.5	0.0092	Upregulation
RAC1	Rac family small GTPase 1	4.3	0.0044	Upregulation
MAPK10	Mitogen-activated protein kinase 10	4.3	0.0049	Upregulation
BCL3	BCL3 transcription coactivator	4.1	0.0032	Upregulation
CSF3	Colony stimulating factor 3	3.9	0.0016	Upregulation
SCAMP3	Secretory carrier-associated membrane protein 3	3.8	0.0026	Upregulation
ZBED6	Zinc finger, BED-type containing 6	3.7	0.0072	Upregulation
LRRC40	Leucine-rich repeat-containing protein 40	3.5	0.0032	Upregulation
CCL21	Chemokine (C-C motif) ligand 21	3.5	0.0045	Upregulation
MTIF2	Mitochondrial translational initiation factor 2	-15.6	0.0056	Downpregulation
MFSD7	Major facilitator superfamily domain-containing protein 7	-15.1	0.0061	Downpregulation
PSG8	Pregnancy specific beta-1-glycoprotein 8	-14.3	0.0043	Downpregulation
GLTPD	Glycolipid transfer protein domain containing 1	-14.1	0.0065	Downpregulation
LYZL2	Lysozyme like 2	-13.8	0.0095	Downpregulation
CDH1	Cadherin 1	-13.2	0.0054	Downpregulation
HDAC1	Histone deacetylase 1	-12.6	0.0044	Downpregulation
EXO1	Exonuclease 1	-12.5	0.0082	Downpregulation
ALDH1L1	Aldehyde dehydrogenase 1 family member L1	-12.2	0.0056	Downpregulation
ACCN1	Amiloride-Sensitive cation channel neuronal 1	-11.8	0.0043	Downpregulation
PSRC1	Proline and serine rich coiled-coil 1	-11.3	0.0067	Downpregulation
BAX	BCL2 associated X Protein	-10.9	0.0017	Downpregulation
SORT1	Sortilin 1	-10.7	0.0016	Downpregulation
SYPL2	Synaptophysin like 2	-10.7	0.0034	Downpregulation

E2F2	E2F transcription factor 2	-10.5	0.0015	Downpregulation
FOXP3	Forkhead box P3	-10.4	0.0066	Downpregulation
ZFP90	ZFP90 zinc finger protein	-10.3	0.0092	Downpregulation
IKZF4	IKAROS family zinc finger 4	-10.2	0.0044	Downpregulation
GLI1	GLI family zinc finger 1	-9.8	0.0033	Downpregulation
EGR4	Early growth response 4	-9.5	0.0054	Downpregulation
LRG1	Leucine-rich alpha-2-glycoprotein 1	-9.2	0.0036	Downpregulation
PFDN6	Prefoldin subunit 6	-8.8	0.0092	Downpregulation
INTS12	Integrator complex subunit 12	-8.5	0.0036	Downpregulation
DHX16	DEAH-box helicase 16	-8.3	0.0072	Downpregulation
AMOTL1	Angiomotin-like protein 1	-7.6	0.0055	Downpregulation
MCDR1	Macular dystrophy, retinal, 1	-7.4	0.0051	Downpregulation
FAR1	Fatty acyl-coA reductase 1	-7.2	0.0083	Downpregulation
GLYAT	Glycine-N-acyltransferase	-7.0	0.0032	Downpregulation
PLCB1	Phospholipase C Beta 1	-6.8	0.0055	Downpregulation
PRDM16	PR/SET domain 16	-6.8	0.0021	Downpregulation
ZFPM1	Zinc finger protein, FOG family member 1	-6.7	0.0014	Downpregulation
DVL2	Dishevelled segment polarity protein 2	-6.5	0.0073	Downpregulation
GRHPR	Glyoxylate and hydroxypyruvate reductase	-6.5	0.0025	Downpregulation
KDM1A	Lysine demethylase 1A	-6.5	0.0052	Downpregulation
SOX13	SRY-Box 13	-6.3	0.0058	Downpregulation
NEAT1	Nuclear paraspeckle assembly transcript 1	-6.2	0.000062	Downpregulation
MCDR1	Macular dystrophy, retinal, 1	-6.0	0.0027	Downpregulation
CDCA7L	Cell division cycle-associated 7-like protein	-5.9	0.0044	Downpregulation
SEH1L	SEH1 like nucleoporin	-5.8	0.0062	Downpregulation
APIP	APAF1 interacting protein	-5.6	0.0024	Downpregulation
TNS3	Tensin 3	-5.6	0.0092	Downpregulation

CHD4	Chromodomain helicase DNA binding protein 4	-5.6	0.0011	Downpregulation
FGF3	Fibroblast growth factor 3	-5.5	0.0015	Downpregulation
CTCF	CCCTC-binding factor	-5.3	0.0043	Downpregulation
RBBP8	RB binding protein 8, endonuclease	-5.3	0.0024	Downpregulation
NOL4	Nucleolar protein 4	-5.2	0.0074	Downpregulation
PLD1	Phospholipase D1	-5.1	0.0038	Downpregulation
ZBTB18	Zinc finger and BTB domain containing 18	-4.6	0.0055	Downpregulation
RTN4	Reticulon 4	-4.3	0.0067	Downpregulation
GUCA1A	Guanylate cyclase activator 1A	-4.1	0.0026	Downpregulation
KIF2A	Kinesin family member 2A	-4.1	0.0012	Downpregulation
ECT2	Epithelial cell transforming 2	-3.7	0.0074	Downpregulation
SV2B	Synaptic vesicle glycoprotein 2B	-3.5	0.0092	Downpregulation
HES7	Hes family BHLH transcription factor 7	-3.5	0.0035	Downpregulation
HDAC2	Histone deacetylase 2	-3.2	0.0062	Downpregulation
CALM2	Calmodulin 2	-3.1	0.0045	Downpregulation

Supplementary Table-7. The FOXP3-associated proteins identified by LC-MS/MS

Protein	Protein description	Molecular weight (kDa)	MASCOT scores
FOXP3	Forkhead Box P3	47	783
HDAC1	Histone Deacetylase 1	55	745
β-Actin	Actin Beta	42	702
CtBP2	C-Terminal Binding Protein 2	49	686
CTLA4	Cytotoxic T-lymphocyte associated protein 4	25	675
GATA3	GATA binding protein 3	48	635
NFATC2	Nuclear factor of activated T cells 2	100	616
TNFRSF18	TNF receptor superfamily member 18	26	603
ITGB8	Integrin subunit beta 8	86	596
GATA3	GATA binding protein 3	48	580
IRAK4	Interleukin 1 receptor associated kinase 4	52	572
HIPK2	Homeodomain interacting protein kinase 2	131	562
IRF1	Interferon regulatory factor 1	37	556
USP7	Ubiquitin specific peptidase 7	128	550
IKZF2	IKAROS Family Zinc Finger 2	60	543
PDCD1	Programmed cell death 1	32	526
ITCH	Itchy E3 Ubiquitin protein ligase	103	520
KLF8	Secreted and transmembrane protein 1	39	515
SMAD4	SMAD Family Member 4	60	512
GPS2	G Protein Pathway Suppressor 2	37	504
GATA1	GATA Binding Protein 1	43	498
USF1	Upstream Transcription Factor 1	34	494
MAPK1	Mitogen-Activated Protein Kinase 1	41	483
EMP2	Epithelial membrane protein 2	19	472
NOD2	Nucleotide binding oligomerization domain containing 2	115	469
CARHSP1	Calcium-regulated heat stable protein 1	16	466
CTRL1	Chymotrypsin-like protease	28	452
PSMB1	Proteasome subunit Beta 1	26	437

PYDC2	Pyrin domain containing 2	11	427
CHD7	Chromodomain helicase DNA binding protein 7	336	419
FGL1	Fibrinogen-like protein 1	36	412
INTS9	Integrator complex subunit 9	74	399
ODF1	Outer dense fiber protein 1	28	384
MFAP2	Microfibrillar-associated protein 2	21	378
GBP2	Guanylate binding protein 2	67	352
GNL2	G protein nucleolar 2	84	345
FPGT	Fucose-1-phosphate guanylyltransferase	68	336
ULK4	UNC-51 like kinase 4	142	315
EAF1	ELL associated factor 1	29	311
GNPDA1	Glucosamine-6-phosphate isomerase 1	33	303
BRAT1	BRCA1-associated ATM activator 1	88	294
ING3	Inhibitor of growth protein 3	47	283
MOSPD3	Motile sperm domain containing 3	26	268
SUMF2	Sulatase-modifying factor 2	34	257
NRBF2	Nuclear receptor-binding factor 2	32	251
CCDC70	Coiled-coil domain-containing protein 70	29	245
WBP4	WW domain-binding protein 4	43	237
DDX23	DEAD-box helicase 23	96	225
EMP1	Epithelial membrane protein 1	18	215
LRRK2	Leucine-rich repeat kinase 2	286	206
PDCD7	Programmed cell death protein 7	55	199
CHSY1	Chondroitin sulfate synthase 1	92	192
SCAMP5	Secretory carrier-associated membrane protein 5	26	183
ELL3	Elongation factor RNA polymerase II-like 3	45	177
CSDC2	Cold shock domain-containing protein D2	17	162
NOL12	Nucleolar protein 12	25	155
THAP7	THAP domain-containing protein 7	34	143
PI4KA	Phosphatidylinositol 4-kinase alpha	237	104

Supplementary Table-8. Potential targets of miR-5688 and miR-95-5p in ALI

miRNAs	miRNA sequence	Target gene	Target region (3'-UTR)
miR-95-5p	UCAAUAAAUGUCUGUUGAAUU	TGBF1	TTTATTGA (308-315)
miR-5688	UAACAAACACCUGUAAAACAGC	IL1B	GTTTGTT (362-368)
